# Patterns of Mass Mortality among Rocky Shore Invertebrates across 100 km of Northeastern Pacific Coastline

**DOI:** 10.1371/journal.pone.0126280

**Published:** 2015-06-03

**Authors:** Laura J. Jurgens, Laura Rogers-Bennett, Peter T. Raimondi, Lauren M. Schiebelhut, Michael N. Dawson, Richard K. Grosberg, Brian Gaylord

**Affiliations:** 1 Bodega Marine Laboratory and Department of Evolution and Ecology, University of California at Davis, Bodega Bay, California, United States of America; 2 California Department of Fish and Wildlife and the Wildlife Health Center, University of California at Davis, Bodega Marine Laboratory, Bodega Bay, California, United States of America; 3 Department of Ecology and Evolution, University of California at Santa Cruz, Santa Cruz, California, United States of America; 4 School of Natural Sciences, University of California at Merced, Merced, California, United States of America; 5 Department of Evolution and Ecology, University of California at Davis, Davis, California, United States of America; The Evergreen State College, UNITED STATES

## Abstract

Mass mortalities in natural populations, particularly those that leave few survivors over large spatial areas, may cause long-term ecological perturbations. Yet mass mortalities may remain undocumented or poorly described due to challenges in responding rapidly to unforeseen events, scarcity of baseline data, and difficulties in quantifying rare or patchily distributed species, especially in remote or marine systems. Better chronicling the geographic pattern and intensity of mass mortalities is especially critical in the face of global changes predicted to alter regional disturbance regimes. Here, we couple replicated post-mortality surveys with preceding long-term surveys and historical data to describe a rapid and severe mass mortality of rocky shore invertebrates along the north-central California coast of the northeastern Pacific Ocean. In late August 2011, formerly abundant intertidal populations of the purple sea urchin (*Strongylocentrotus purpuratus*, a well-known ecosystem engineer), and the predatory six-armed sea star (*Leptasterias* sp.) were functionally extirpated from ~100 km of coastline. Other invertebrates, including the gumboot chiton (*Cryptochiton stelleri*) the ochre sea star (*Pisaster ochraceus*), and subtidal populations of purple sea urchins also exhibited elevated mortality. The pattern and extent of mortality suggest the potential for long-term population, community, and ecosystem consequences, recovery from which may depend on the different dispersal abilities of the affected species.

## Introduction

Mass mortalities have the capacity to drive persistent ecosystem changes [1–2]. The magnitude and duration of the changes may be determined in part by the spatial extent and intensity of mortality, and also by the ecological roles and life-history attributes of the species affected [3]. For instance, in marine systems, fecundity, pelagic larval duration, reproductive season, and local density may interact with patterns of ocean circulation and dispersal to accelerate or retard rates of recovery through their effects on population growth or immigration from nearby locations. Such complexities reinforce the need to carefully chronicle the geographic pattern and intensity of mass mortalities with sufficient resolution to track their trajectories and consequences in the face of global changes that are predicted to alter regional disturbance regimes [4–5].

The challenges to understanding ecological consequences of severe perturbations are substantial. Common drivers of mass mortalities, including storms, heat waves, disease epidemics, and harmful algal blooms (HABs) are unpredictable [6–10]. Some fraction of mass mortality events likely goes unnoticed or is under-reported. Other events may be reported but suffer from a paucity of data regarding their degree of severity or level of spatial coherence. These challenges may apply especially strongly in the ocean, where baseline data describing species occurrences and population sizes are often scarce. Even along highly populated coastlines, such as California (USA), reports of at least two mass mortality events in highly visible near-shore species (an intertidal sea urchin and a canopy-forming kelp) remain limited to “personal communications” or brief historical notes [11–15].

Such incomplete information limits our ability to detect trends in the frequency and intensity of mortality events, both of which are predicted to increase under global change. Documentation exists for a suite of large-scale, high-severity mortality events in benthic systems, enabling assessment of at least some of their long-term ecological impacts. For example, it is known that declines of the eelgrass *Zostera marina* caused basin-wide extinction of the limpet *Lottia alveus* [16] and acute reductions in eastern populations of Brant’s goose (*Branta bernicla*) [17]. Widespread mortality of long-spined sea urchins (*Diadema antillarium*) allowed overgrowth of coral reefs by fleshy algae and thus prolonged perturbation of Caribbean ecosystems [18–20]. In contrast, less-severe disturbances that impose localized or spatially patchy effects, and/or that are characterized by greater baseline survivorship, may not receive as much attention. They can also have more moderate or shorter-duration impacts due to the increased likelihood of recovery by immigration or local reproduction [9]. Since benthic mass mortalities were last reviewed, predominantly covering events before 1999 [7, 21, 22], documented die-offs have tended to be either localized or have had patchy effects characterized by areas of substantial survivorship (Table 1; [23–42]).

**Table 1:** Mass mortality events of benthic marine species occurring since 2000.

**Year(s), Location**	**Affected organisms**	**Suspected cause(s)**	**Mortality range**	**Spatial extent (km^2^)^a^^,^^b^**	**Spatial pattern reported**	**References**
2001–2003, Ligurian coast, N Mediterranean	Zoanthid (*Parazoanthus axinellae*)	Disease, High water temperature	~ 90%	0.0001	ND	[23]
2003, N Mediterranean	Gorgonians, sponges, bryozoans, bivalves; (multiple species)	High water temperature	5–80% *	1500	patchy	[24]
2003, Canary Islands, SE Atlantic	Sea urchin (*Paracentrotus lividus*)	Disease, high water temperature	0–95%	50	patchy	[25]
2003, 2009, Nova Scotia, NW Atlantic	Sea urchin (*Strongylocentrotus droebachiensis)*	Disease, hurricanes	0–100%	3	patchy	[26]
2004–2005, Cape Cod, NW Atlantic	Sea scallop (*Placopecten magellanicus*)	Unknown	35%	4000	ND	[27]
2005, Great BarrierReef, Coral Sea	Corals (multiple species)	Solar radiation, low tide exposure	10–40% *	10	patchy	[28]
2005, Florida, E Gulf of Mexico	Fishes, sponges (multiple species) Coral (*Cladocora arbuscula*)	Algal bloom, hypoxia	Sponges:6–7%; other taxa: ND	10	ND	[29]
2005–2007, Caribbean Sea	Corals (multiple species)	High water temperature, disease	0–70%	2 x 10^6^	patchy	[30]
2008, Coliumo Bay, Chile, SE Pacific	Crabs, fishes (multiple species per taxon)	Hypoxia	~90%	5	ND	[31, 32]
2008, Sardinia, N Mediterranean	Octocoral (*Paramuricea clavata*)	Disease, high water temperature	0–100% *	2	ND	[33]
2008, 2009, N Mediterranean	Sponges (*Ircinia* spp.)	Disease, high water temperature	0–95% *	700	patchy	[34–36]
2009, Isla Natividad, Mexico, NE Pacific	Pink abalone (*Haliotis corrugata)*	Hypoxia	41%	10	ND	[37]
2009, Bahia de Huatulco, NE Pacific	Sea urchin (*Diadema mexicanum*)	ND	100%	0.001	ND	[38]
2010, Florida Keys, Straits of Florida	Corals (multiple species)	Low water temperature	17–100%	0.01	ND	[39]
2010, 2011, Malibu, California, NE Pacific	Sea urchin (*Strongylocentrotus purpuratus*)	Low salinity, sediment	0–99%	0.01	patchy	[40]
**2011, Sonoma county, California, NE Pacific**	**Sea urchin (*S*. *purpuratus*), sea star (*Leptasterias* sp.)**	**Harmful algal bloom toxicity**	**>99.99%**	**100**	**continuous**	**this study**
2012, Comau Fjord, Chile, SE Pacific	Coral (*Desmophyllum dianthus*)	Methane and/or sulfide seeps, hypoxia	50–99%	8.4	ND	[41]
2013–present, West coast of N America, NE Pacific	Sea stars (multiple species)	Wasting disease	0–70%	5000	patchy	[42]
2001–2003, Ligurian coast, N Mediterranean	Zoanthid (*Parazoanthus axinellae*)	Disease,High water temperature	~ 90%	0.0001	ND	[23]
2003, N Mediterranean	Gorgonians, sponges, bryozoans, bivalves; (multiple species)	High water temperature	5–80% *	1500	patchy	[24]
2003, Canary Islands, SE Atlantic	Sea urchin (*Paracentrotus lividus*)	Disease, high water temperature	0–95%	50	patchy	[25]
2003, 2009, Nova Scotia, NW Atlantic	Sea urchin (*Strongylocentrotus droebachiensis)*	Disease, hurricanes	0–100%	3	patchy	[26]
2004–2005, Cape Cod, NW Atlantic	Sea scallop (*Placopecten magellanicus*)	Unknown	35%	4000	ND	[27]
2005, Great BarrierReef, Coral Sea	Corals (multiple species)	Solar radiation, low tide exposure	10–40% *	10	patchy	[28]
2005, Florida, E Gulf of Mexico	Fishes, sponges (multiple species) Coral (*Cladocora arbuscula*)	Algal bloom, hypoxia	Sponges:6–7%; other taxa: ND	10	ND	[29]
2005–2007, Caribbean Sea	Corals (multiple species)	High water temperature, disease	0–70%	2 x 10^6^	patchy	[30]
2008, Coliumo Bay,Chile, SE Pacific	Crabs, fishes (multiple species per taxon)	Hypoxia	~90%	5	ND	[31, 32]
2008, Sardinia, N Mediterranean	Octocoral (*Paramuricea clavata*)	Disease, high water temperature	0–100% *	2	ND	[33]
2008, 2009, N Mediterranean	Sponges (*Ircinia* spp.)	Disease, high water temperature	0–95% *	700	patchy	[34–36]
2009, Isla Natividad, Mexico, NE Pacific	Pink abalone (*Haliotis corrugata)*	Hypoxia	41%	10	ND	[37]
2009, Bahia de Huatulco, NE Pacific	Sea urchin (*Diadema mexicanum*)	ND	100%	0.001	ND	[38]
2010, Florida Keys, Straits of Florida	Corals (multiple species)	Low water temperature	17–100%	0.01	ND	[39]
2010, 2011, Malibu, California, NE Pacific	Sea urchin (*Strongylocentrotus purpuratus*)	Low salinity, sediment	0–99%	0.01	patchy	[40]
**2011, Sonoma county, California, NE Pacific**	**Sea urchin (*S*. *purpuratus*), sea star (*Leptasterias* sp.)**	**Harmful algal bloom toxicity**	**>99.99%**	**100**	**continuous**	**this study**
2012, Comau Fjord, Chile, SE Pacific	Coral (*Desmophyllum dianthus*)	Methane and/or sulfide seeps, hypoxia	50–99%	8.4	ND	[41]
2013–present, West coast of N America, NE Pacific	Sea stars (multiple species)	Wasting disease	0–70%	5000	patchy	[42]

Note that the references provided represent to our knowledge the original report(s) describing events in wild populations, and do not include subsequent follow-up publications focused on the same events. Events are summarized from a review of 897 articles; see S1 File for a full description of the literature review methods.

*Denotes mortality of colonial species reported as a percentage of affected colonies with partial necrosis, rather than absolute mortality.

ND, no data.

^a^Where not stated explicitly, we estimated spatial extent of study regions from maps or text descriptions.

^b^Note that most published studies do not include the spatial boundaries of mortality (i.e., the geographic locations past which no mortality was observed). When this information was absent, we report here the spatial extent of the study region.

**Fig 1:**
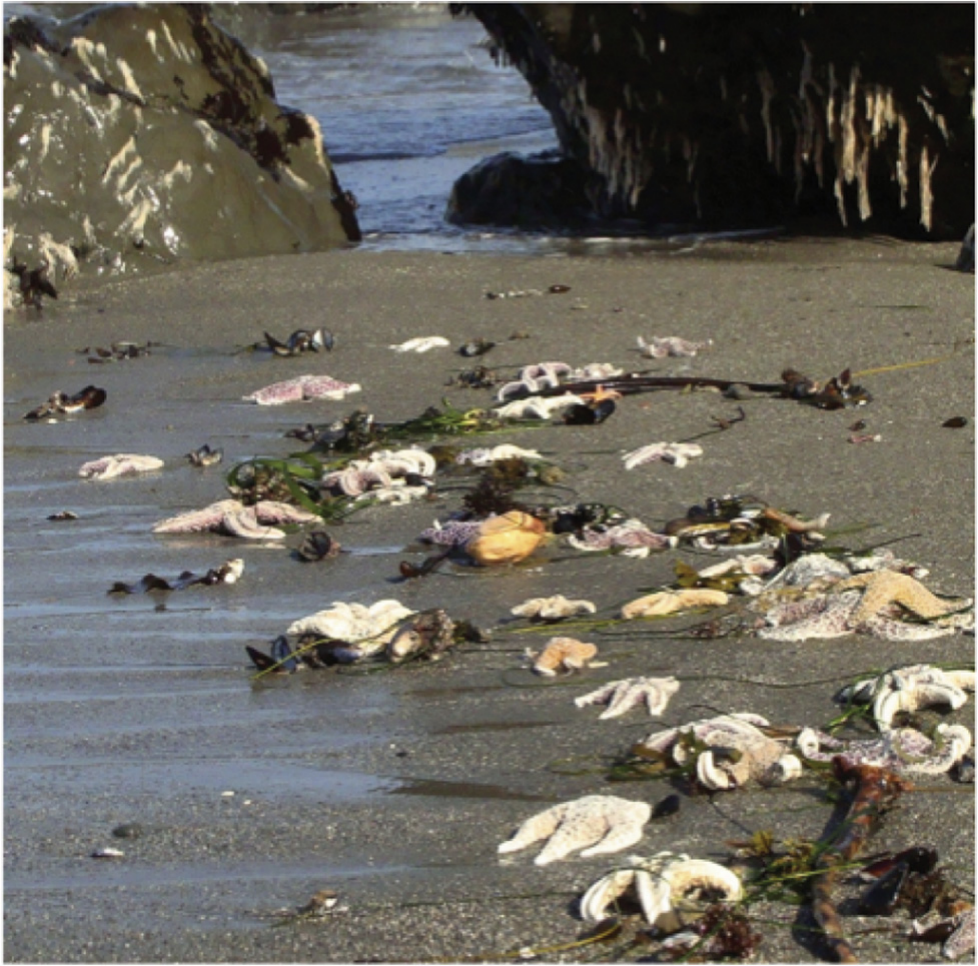
Photograph of dead sea stars. Ochre sea star (*Pisaster ochraceus*) carcasses washed up en masse in late August 2011. Photo credit: Ashley Robart.

Here, we quantify the intensity and spatial pattern of a mass mortality event that had severe to moderate effects on multiple marine invertebrate species over a sharply demarcated region. Elevated mortality first became apparent when thousands of invertebrates held in well-aerated, flow-through seawater tanks died precipitously at the Bodega Marine Laboratory (BML; Bodega Bay, California, USA) between August 28 and 30, 2011. More than 99% of captive purple sea urchins (*S*. *purpuratus* [n > 1000]; LJJ *pers*. *obs*.), 100% of six-armed sea stars (*Leptasterias* spp. [n = 50]; S. Gravem, *pers*. *comm*.) and several ochre sea stars (*Pisaster ochraceus*) died (LJJ, *pers*. *obs*.). Concurrently, tens of thousands of purple urchins also disappeared from a commonly used scientific collection site in Sea Ranch, California, USA (LJJ, *pers*. *obs*.). Carcasses of heavy-bodied invertebrates, including large adult *P*. *ochraceus*, gumboot chitons (*Cryptochiton stelleri*) and red abalone (*Haliotis rufescens*) washed up on beaches (Fig 1). Motivated by these early observations, we proceeded to characterize the severity and geographic pattern of mortality in the regional populations of *S*. *purpuratus*, *Leptasterias* sp., *P*. *ochraceus*, and *C*. *stelleri* across the region. We also investigated potential effects on another, rarer sea star, *Henricia* sp. This work was conducted with the goal of providing a foundation for understanding the ecological effects of the die-off and any succeeding dynamics of population recovery.

## Materials and Methods

### Study region and species

**Fig 2:**
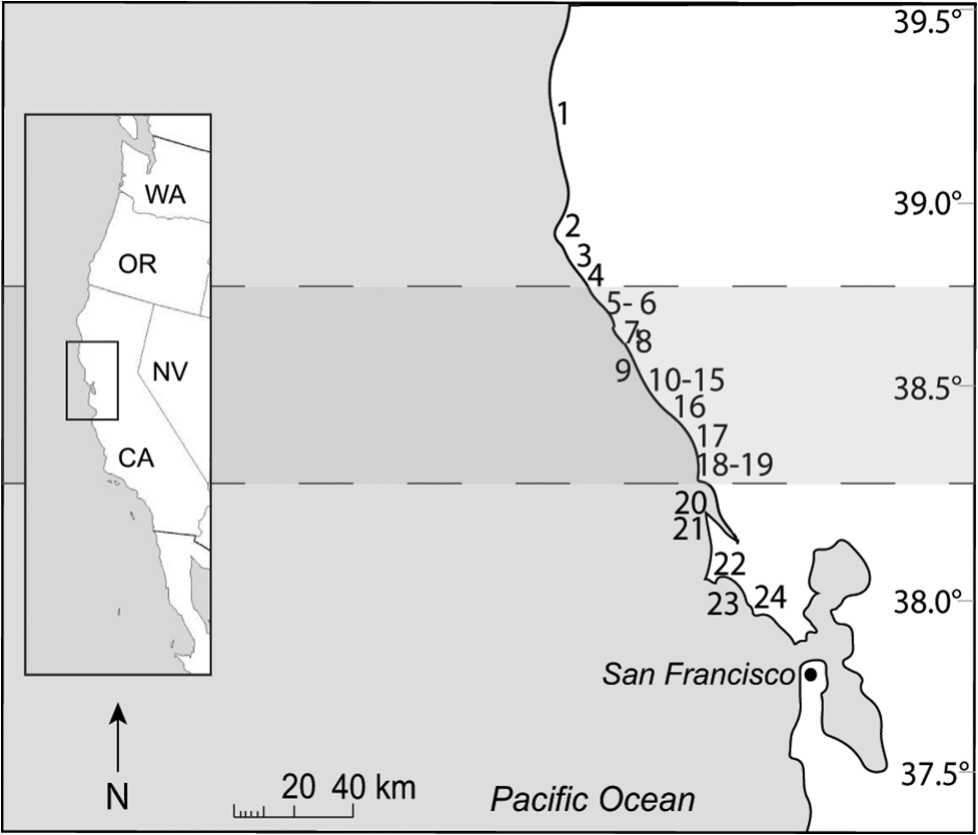
Map of the study region (north-central California, USA). The region between dashed lines marks the zone of invertebrate mortality, which spanned approximately 100 km of coastline (fractal unit 0.1 km). Sites are numbered north to south from Van Damme State Park (Site 1, 39.16° N) to Duxbury Reef (Site 24, 37.53° N). See S1 Table for GPS locations and details of surveys conducted at each location.

The study area spans 270 kilometers of relatively inaccessible coastline, extending from 39°16' N in Mendocino County to the southern edge of the Point Reyes peninsula at 37°53' N, California (Fig 2). Approximately 75% of this stretch is steep, wave-exposed rocky shore. The remainder consists of isolated sandy beaches and small estuaries. The region falls within a productive coastal upwelling zone in which surface waters are seasonally infused with deep-water nutrients, fueling local food webs and fostering a high diversity of rocky shore invertebrates [43, 44], while also facilitating microalgal blooms [45, 46].

Four of the focal taxa tracked in this study (*S*. *purpuratus*, *Leptasterias* sp., *P*. *ochraceus*, *C*. *stelleri*) were historically widespread and ecologically important in the study area [47–50]. The region includes a central portion of the purple urchin’s range (Alaska to Baja California [51–53]), and at many locations within the study region, purple urchins were documented as the most abundant intertidal macroinvertebrate [47], inhabiting large swaths of the low intertidal zone where densities could exceed 600 individuals per m^2^ [50]. While not nearly so abundant, *P*. *ochraceus* also can be common in the region. The ochre sea star is a large-bodied (mean wet mass ~450 g [54]), well-studied keystone predator, particularly of mussels, from Alaska to south-central California [55, 56]. Numbers of ochre stars are currently declining, due to a sea star wasting disease along the U.S. west coast, but symptoms of this disease were not observed until June 2013, nearly two years after the event we describe here [42]. Small, often cryptic, six-armed sea stars of the genus *Leptasterias* (in this region, most often of the *L*. *hexactis* form; mean wet mass ~5 g; [54]) also range from Alaska to central California [57]. We observed only the *L*. *hexactis* morphotype in this study and preliminary DNA sequencing by our group suggests a single species (S1 File), but there are ongoing questions about divergence and speciation in *Leptasterias* [57–59]. Six-armed sea stars are important predators of small mollusks and have been historically common across the study region [48], [60–62]. The gumboot chiton, *Cryptochiton stelleri*, is the largest invertebrate herbivore in the ecosystem, reaching 33 cm in length and inhabiting low intertidal and shallow subtidal habitats from California to Alaska and Japan [63]. An unexplained localized die-off of *Leptasterias* sp. and *S*. *purpuratus* living in mid-to-high intertidal areas occurred in 2010 at Bodega Marine Reserve (site 18; S. Gravem, E. Sanford and J. Sones, *pers*. *comms*.). *Leptasterias* sp. remained common at adjacent locations (sites 16, 17; LJJ, *pers*. *obs*.) and *S*. *purpuratus* remained abundant in low intertidal habitats at the affected site as of July 2011, prior to the event we describe here (LJJ, *pers*. *obs*.).

### Intertidal purple urchin mortality

**Fig 3:**
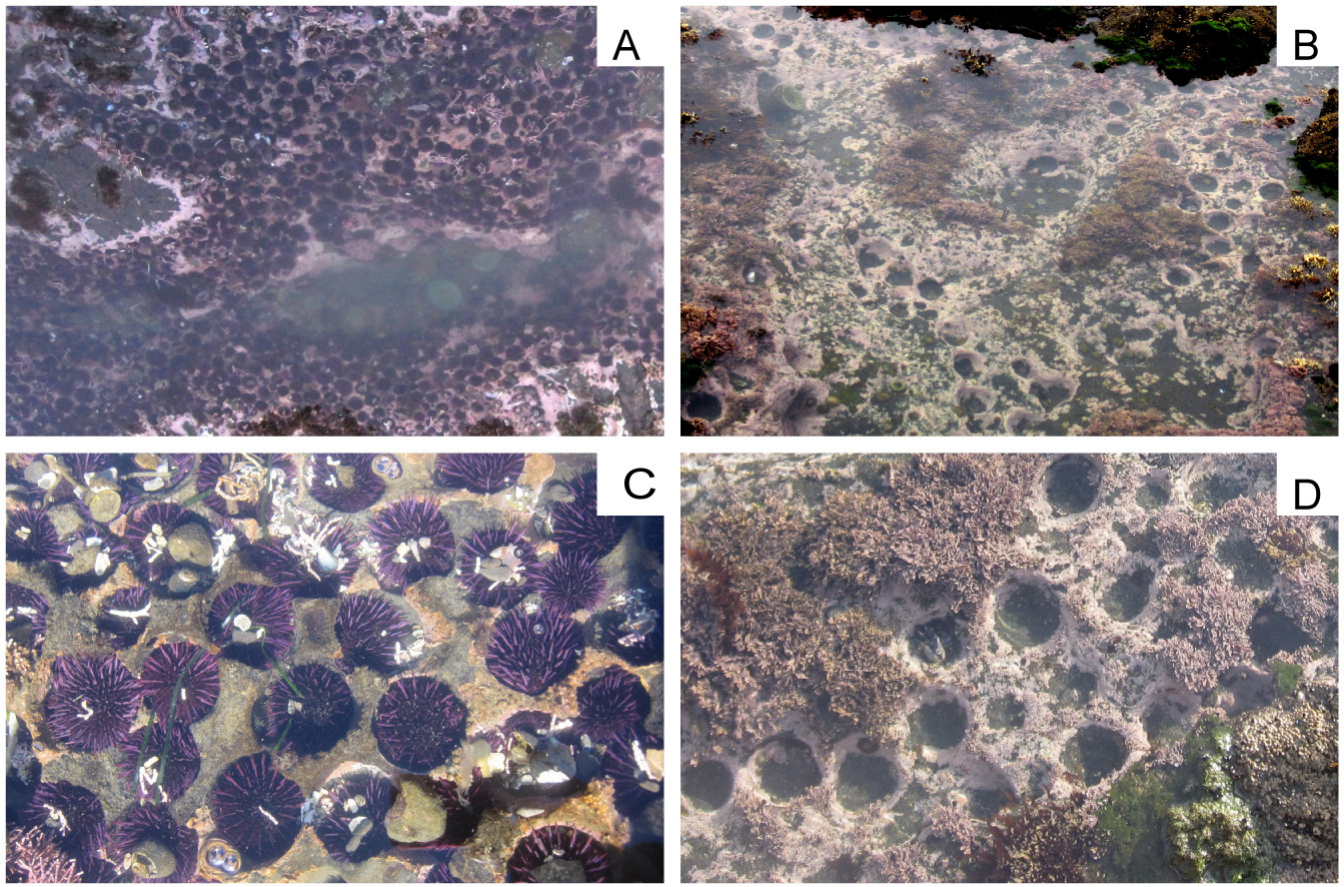
Purple urchin burrows in bedrock, with and without occupants. (A) A large tidepool outside the impact zone (site 4), with a dense population of live *Strongylocentrotus purpuratus* in November 2011. (B) Empty urchin burrows in a tidepool (site 16) after the mass mortality event. (C) Close-up of occupied purple urchin burrows, showing one urchin per burrow and 100% occupancy; note urchins are covered with debris. (D) Close-up of clean, empty urchin burrows such as those used to estimate pre-event densities.

We quantified the severity of intertidal purple urchin mortality based on their unique habitat-modifying fingerprint in intertidal environments. In this region of extreme, wave-swept shores, urchins excavate and occupy individual burrows in the bedrock [48, 64, 65] (Fig 3). No other species in the ecosystem makes similar modifications to rock. Grazing activities and constant abrasion from moving spines maintain clean burrows while urchins occupy them, but sessile invertebrates such as barnacles, spirorbid worms and anemones rapidly colonize vacated burrows [66]. A clean, empty urchin burrow with no overgrowth by sessile species therefore provides evidence of recent (i.e., within weeks) urchin habitation (Fig 3B and 3D). We used this distinctive indicator to estimate pre-event urchin populations, calculating pre-event density as the combined density of live urchins plus clean, empty burrows. We calibrated this quantity using the average occupancy rate of burrows at four control sites (sites 1, 2, 23, 24; Fig 2; S1 Table). This calibration did not change our initial estimates since burrow occupancy rate across all control sites was 100% (SD = 0.01).

In our analysis of the severity of intertidal purple urchin mortality, we compared pre-event and post-event urchin densities at 11 sites (Fig 2; see S1 Table for detailed locations). At five of these (sites 8, 10, 14, 16, 18; Fig 2), we had recorded robust urchin populations between May and July 2011, prior to the onset of laboratory mortality on August 28, 2011. Initial scouting of the same sites between August 31 and September 10, 2011 suggested a complete loss of these populations, although comparatively high low tides and large waves hampered full surveys. We then began quantitative mortality surveys at four of these sites that spanned the extent of the study region (37° 53’ N to 39° 16’ N; sites 1, 14, 16, 24; Fig 2, S1 Table) between September 26 and 30, 2011, once tides became low enough to access urchin habitat. We completed our first round of population surveys at the remainder of our 11 core sites during the subsequent three accessible low tide series, between October and December 2011.

Since local populations typically comprise hundreds of thousands of animals, unevenly distributed across topographically complex habitat [47], we used mean density within established urchin assemblages rather than absolute abundance to assess population impacts at each site. We counted all live purple urchins and clean, empty urchin burrows in twelve 0.25 m^2^ quadrats placed randomly within established urchin habitats at each site. We used a Fisher’s Exact Test to compare densities by site, since differences violated assumptions of more complex approaches (e.g., zero-inflation and spatial continuity of impacted sites).

### Subtidal purple urchin mortality

Comparatively few purple urchins inhabit deeper subtidal depths in this region, where they tend to be replaced by the larger red urchin, *Mesocentrotus* (formerly *Strongylocentrotus*) *franciscanus*, especially at depths > 8 m [47, 67]. However, survivors in subtidal habitats could be important facilitators of recovery if post-event densities remained sufficient for successful reproduction, or if individuals aggregated in groups (urchins are dioecious broadcast spawners and successful fertilization requires that males and females be in close proximity [65, 68–70]). To estimate subtidal-zone mortality and assess survivor density, we compared purple urchin densities in diver surveys conducted before (2005–2010) and after (2012) the die-off at four sites in the study region (sites 11–13, 15; Fig 2; S1 Table). The surveys were part of regular monitoring efforts conducted between July and October of each year (led by LRB for California Department of Fish and Wildlife). Pairs of divers counted urchins along 30 x 2 m^2^ transects in rocky reef habitats in three depth classes at each site (0–9 m, 9.1–13.7 m and 13.8–18.3 m). Transects were placed at pre-selected random GPS coordinates within each depth stratum, and new random coordinate locations were generated for each survey year. At each site, divers initiated 18 transects in the shallow depth stratum and 9 transects in each of the two deeper strata. Ocean conditions impacted the number of transects completed at each site in any given survey year. Transects frequently contained zero urchins and we used log transformations of density data for all analyses. Because we were interested in differences between pre-event and post-event subtidal-zone densities, as well as differences within each depth stratum (rather than an interaction effect), and because the number of transects sampled varied by depth, we used *t-*tests to examine density changes for the pooled data at each depth stratum individually.

### Urchins as indicators of the spatial scale of disturbance

Once we determined the severity of intertidal purple urchin mortality at our 11 core sampling sites, we added nine sites to increase spatial resolution, to search more extensively for survivors, and to more precisely delineate the spatial distribution of mortality ("Fine-grained surveys," S1 Table). Between October 2011 and December 2012, we conducted site-wide searches for purple urchins within and outside the general impact zone across a total of 20 intertidal locations (sites 1–10, 14, 16–24; Fig 2; S1 Table). At each location, we searched a minimum of two and a maximum of 12 person-hours, until we either examined the entire site or counted more than 1000 adult urchins. We searched for live adult urchins and for clean, empty urchin burrows (as indicators of recent mortality). We focused on adults, defined as individuals with test diameters greater than 25 mm [71], since smaller juvenile urchins could in theory have settled after the mortality event. To maximize the probability of detecting survivors given the extreme levels of mortality, we also solicited reports of live purple urchins from other researchers and from coastal residents via local print and radio announcements.

### Population impacts to other invertebrate species

To examine potential impacts to regional populations of *C*. *stelleri*, *Leptasterias* sp., and *P*. *ochraceus*, we compared all available monitoring data collected between 2001 and 2011, focusing on data collected in the three years (2008–2010) prior to the mortality event for quantitative comparison with post-event surveys at the same sites (S1 Table). Intertidal-zone monitoring teams surveyed the region prior to the event during baseline assessments for implementation of the California Marine Life Protection Act, and as part of a regular monitoring program (MARINe west coast monitoring: http://www.pacificrockyintertidal.org, led by PTR). Pre-event monitoring efforts provided counts of individuals in 0.25 m^2^ quadrats (n = 18 to 34) placed randomly across three shore elevations at each site. Since the target species are not typically found in the high-shore elevation category used in these surveys, we included only data from quadrats in the middle and lowest shore elevations in our analyses (n = 12 to 22 per site). These surveys were designed to characterize biodiversity and community structure, rather than provide detailed population estimates for these particular species, so total spatial coverage was relatively low (3 to 5.5 m^2^ over sites often exceeding 5000 m^2^), creating a potential for false zeros or overestimation of density when one or two individuals occurred in few total quadrats. To avoid this problem in post-event surveys using the same method, we increased replication (n = 53 to 210 quadrats per site, depending on the amount of suitable habitat) and targeted only species-appropriate shore elevations, leading to a more intense post-event sampling effort. To reduce bias in quantitative comparisons of post-event versus pre-event quadrat densities due to increased sampling effort, we also compared changes in presence versus absence at each location where target species were found before the mortality event.

Early observations during post-event data collection suggested potential effects on sea stars of the genus *Henricia* (we observed only the *H*. *pumila* form [72], and preliminary DNA sequencing by our group suggests a single species (S1 File), although there remain questions about systematics in this genus [72, 73]). We included searches for the comparatively rare *Henricia* sp. in subsequent quadrat surveys that we conducted between late 2012 and early 2014, during which we added additional sites to the north and south of the affected region (S4 Table).

*Cryptochiton stelleri* was rarely detected in pre-event quadrats, likely because sampling did not extend into the lowest intertidal shore elevations preferred by the species. We therefore limited our analysis to post-event data, which we examined for spatial patterns of decreased abundance or absence of *C*. *stelleri* within the impact zone delineated by the loss of large intertidal purple urchin populations. Because *P*. *ochraceus* and *C*. *stelleri* are both large-bodied and conspicuous, but often occur at low densities, we conducted broad-scale swath transects to further estimate post-event densities for these two species. Swath transects covered 831 to 3736 m^2^, depending on the amount of suitable habitat at each site (see S1 File for detailed description).

Complementary studies by others [74, 75] indicated moderate to severe impacts on two subtidal, fished species: the red sea urchin (*M*. *franciscanus*; 50 to 90% mortality at depths less than 20 m) and red abalone (25% estimated mortality). Because these effects were reported previously, we did not attempt to describe them in any further detail.

### Assessing biotic and abiotic conditions coincident with mortality

There were no obvious physical stressors (e.g., a storm, heavy rainfall event, or heat wave) that occurred during late August and early September 2011, when the onset of mortality was observed. Nevertheless, we inspected climatic and oceanographic data to assess the potential for anomalies that might indicate either biotic or abiotic stressors. We accessed salinity, air temperature, water temperature, and seawater chlorophyll-a (a common indicator of phytoplankton concentrations) data from sensors located at the shoreline of BML, operated through the Bodega Ocean Observing Node (BOON, http://bml.ucdavis.edu/boon/) for the month of August 2011. We also scanned offshore swell data from the U.S. National Oceanic and Atmospheric Administration’s National Data Buoy Center (station 40613 Bodega Bay, http://www.ndbc.noaa.gov) for any indicators of potentially anomalous wave disturbance.

### Ethics Statement

All data included in this study were collected using non-destructive sampling methods. Several study locations fall within Marine Protected Areas and parks. For each site location, special jurisdictions and relevant permit information are given in S1 Table.

## Results

### Impacts on purple urchin populations

**Fig 4:**
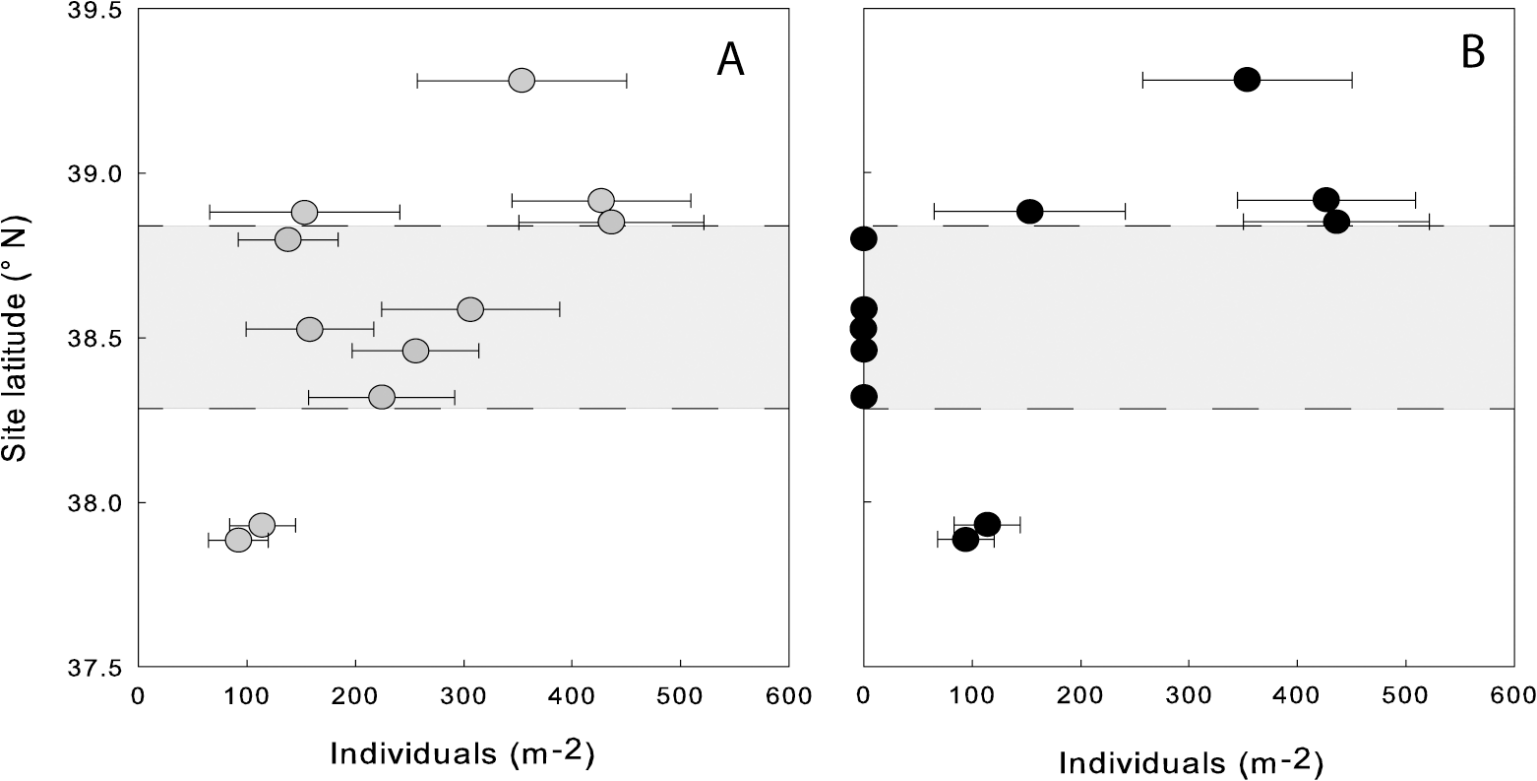
Densities of purple urchins (A) before and (B) after the mass mortality event, by latitude. Bars depict standard deviation across sampled quadrats. Data are from the 11 sites surveyed between September and December 2011 (sites 1–4, 6, 10, 14, 16, 18, 23, 24). The shaded area denotes the impacted region, in which we found zero individuals at all sites surveyed after the event (n = 5).

Purple sea urchins were nearly extirpated from intertidal locations across a contiguous portion of the initial survey range. Pre-event, *S*. *purpuratus* were present at mean densities of 138 to 306 urchins per m^2^ (Fig 4A), but post-event they were absent from the five sites in the center of the survey region (sites 6, 10, 14, 16, 18; Fig 4B). In contrast, we found no evidence for population declines of purple urchins at the six peripheral sites, north of Anchor Bay (site 5) and south of Bodega Head (site 19), where we encountered >1000 individuals within the first 10 minutes of searching and 100% burrow occupancy at all sites with urchin burrows, which indicated no recent density change (sites 1–4, 23, 24; Fig 4A and 4B). These post-event urchin densities were significantly higher outside versus within the impacted region (Fisher's Exact Test, *P* < 0.001).

The fine-grained searches designed to detect any surviving intertidal *S*. *purpuratus* at a broader array of sites confirmed the original estimate of the boundaries of event-related mortality, as well as its uniformity. Across the expanded suite of 20 sites, we documented near-complete mortality inside, and no detectable mortality outside, a contiguous region stretching from site 6 in the north to site 19 in the south (Fig 2). We used this essentially dichotomous pattern of intertidal urchin presence or absence as an operational indicator of the spatial bounds of the event. This impact zone spanned approximately 100 km of coastline (fractal unit 0.1 km).

A total of ten adult *S*. *purpuratus* were found alive intertidally within the impact zone, at sites 7 and 10, between November and December 2012; nine of the ten occurred at Del Mar Landing (site 7), five of which were within 0.25 m of another individual.

**Fig 5:**
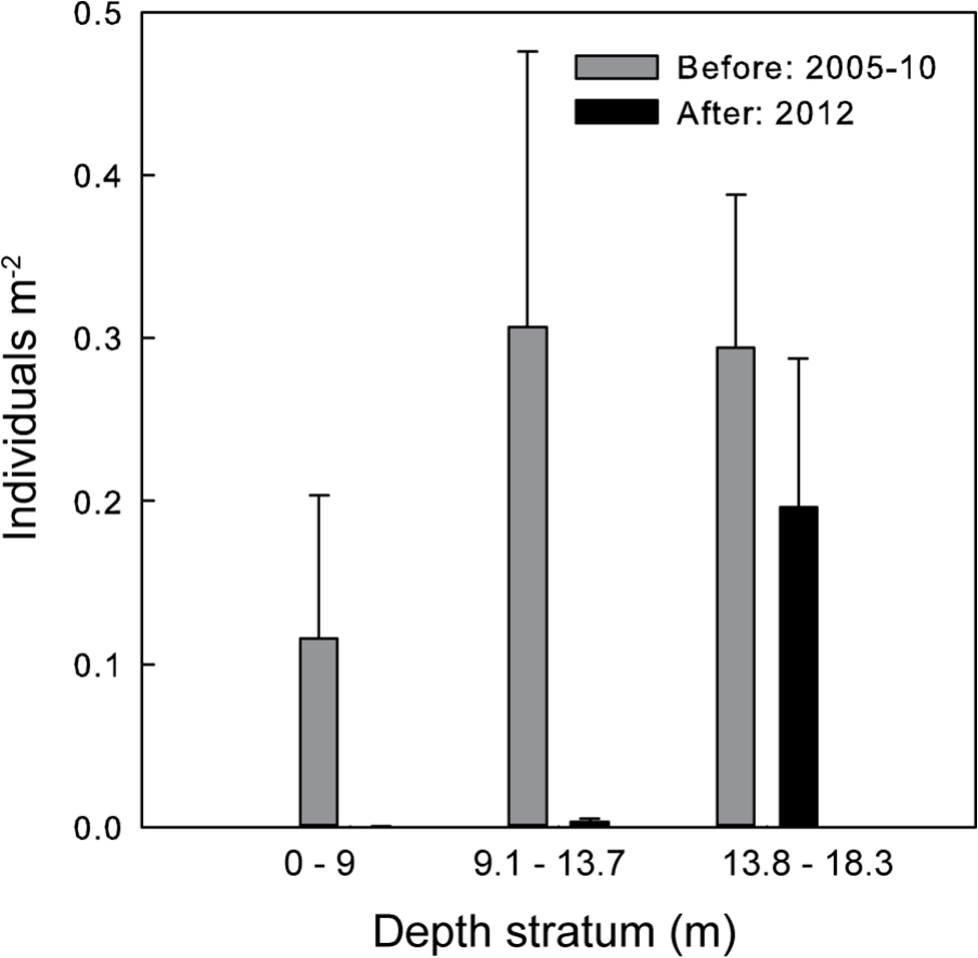
Subtidal-zone densities of purple urchins. Data are shown by depth class, and represent counts (mean ± SE) in transects surveyed before (grey; n = 300) and after (black; n = 146) the mass mortality event. Data from four sites (11–13, 15) in the impact zone are pooled.

Pre-event densities of *S*. *purpuratus* in subtidal zones were already three orders of magnitude smaller than densities in intertidal zones, and appear to have been further reduced, from an average of 0.35 to 0.013 individuals per m^2^ (t_444_ = 2.35, *P* = 0.009). Across the four subtidal-zone sites (11–13, 15), post-event surveys indicated the most severe reductions in purple urchin density occurred in the two shallower depth zones (Fig 5). Mean densities dropped from 0.12 to 0.0002 individuals per m^2^ at depths up to 9 m (t_164_ = 1.79, *P* = 0.038), and from 0.30 to 0.003 individuals per m^2^ in the middle depth zone (9.1 to13.7 m; t_82_ = 2.57, *P* = 0.006). Purple urchin density remained highest in the deepest transects (13.8 to 18.3 m), exhibiting no significant change (t_78_ = 0.82, *P* = 0.21), and averaging 0.2 individuals per m^2^ after the event. The distribution of survivors in the subtidal zone was patchy over the scale of 100 meters (LRB, *pers*. *obs*.).

### Population impacts on other invertebrate species

**Fig 6:**
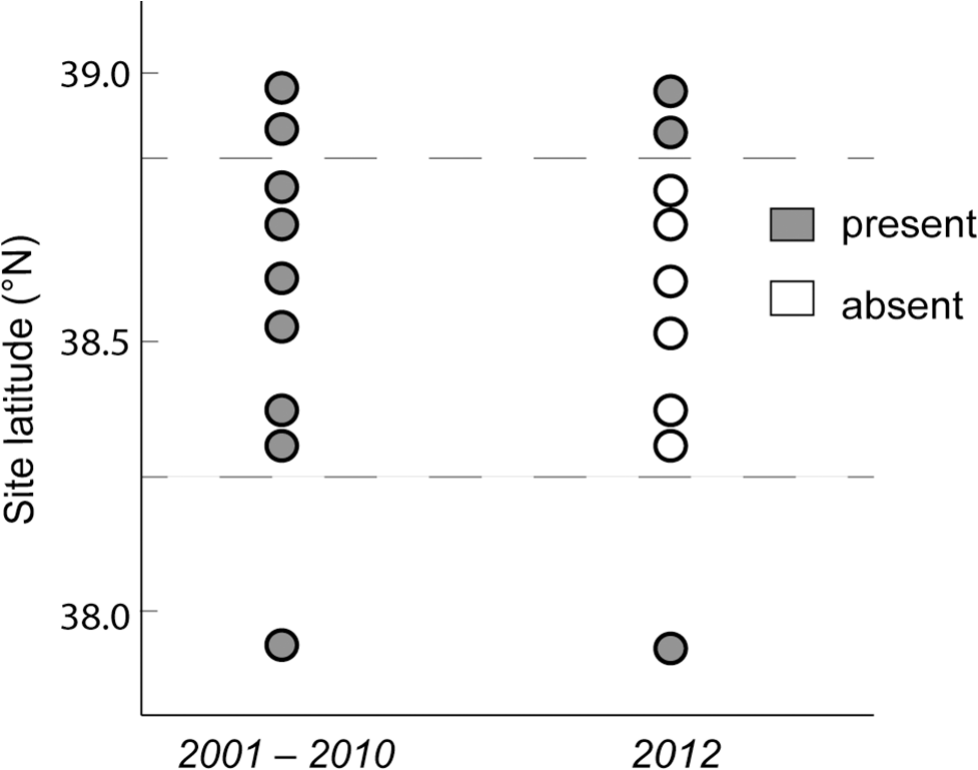
Six-armed sea star presence and absence before and after the mass mortality. Data are shown for surveys before the 2011 mass mortality (2001–2010) and after (2012). In the intervening period, *Leptasterias* sp. became absent from every site within the die-off region in which purple sea urchins experienced mortality (bordered by dashed lines; sites 7, 8, 10, 14, 18, 19). Six-armed sea stars were still found at sites outside the affected region in 2012, as in prior years (sites 3, 4, 24).

We found zero *Leptasterias* sp. at previously occupied sites throughout the 100 km impact zone, indicating widespread extirpation (Fig 6). Densities of six-armed sea stars dropped significantly across the six sites within the impact zone (sites 7, 8, 10, 14, 18, 19), from an average of 3.2 to zero individuals per m^2^ (t_5_ = 4.75, *P* = 0.0025). At sites outside the impact zone (sites 3, 4, 24; Fig 2), we found no significant change in *Leptasterias* sp. density (t_2.6_ = 1.8, *P* = 0.18). Prior to August 2011, *Leptasterias* sp. occurred in every monitoring survey conducted (2001 to 2010) at the six sites within the impact zone, including in the last surveys undertaken before the event (S2 Table). After the event, we did not find a single six-armed star at these six locations, although we increased search areas substantially (from 3–5.5 m^2^ per site to 26–37 m^2^ per site; S2 Table). Similarly, we found zero *Leptasterias* sp. in the large swath transects (covering 831 to 3736 m^2^) at all sites from which purple urchins had disappeared. However, we did find *Leptasterias* sp. at all three previously occupied sites outside the zone of purple urchin loss, in abundances ranging from 2–33 per site (survey areas 13.25–40.25 m^2^; S2 Table).

**Fig 7:**
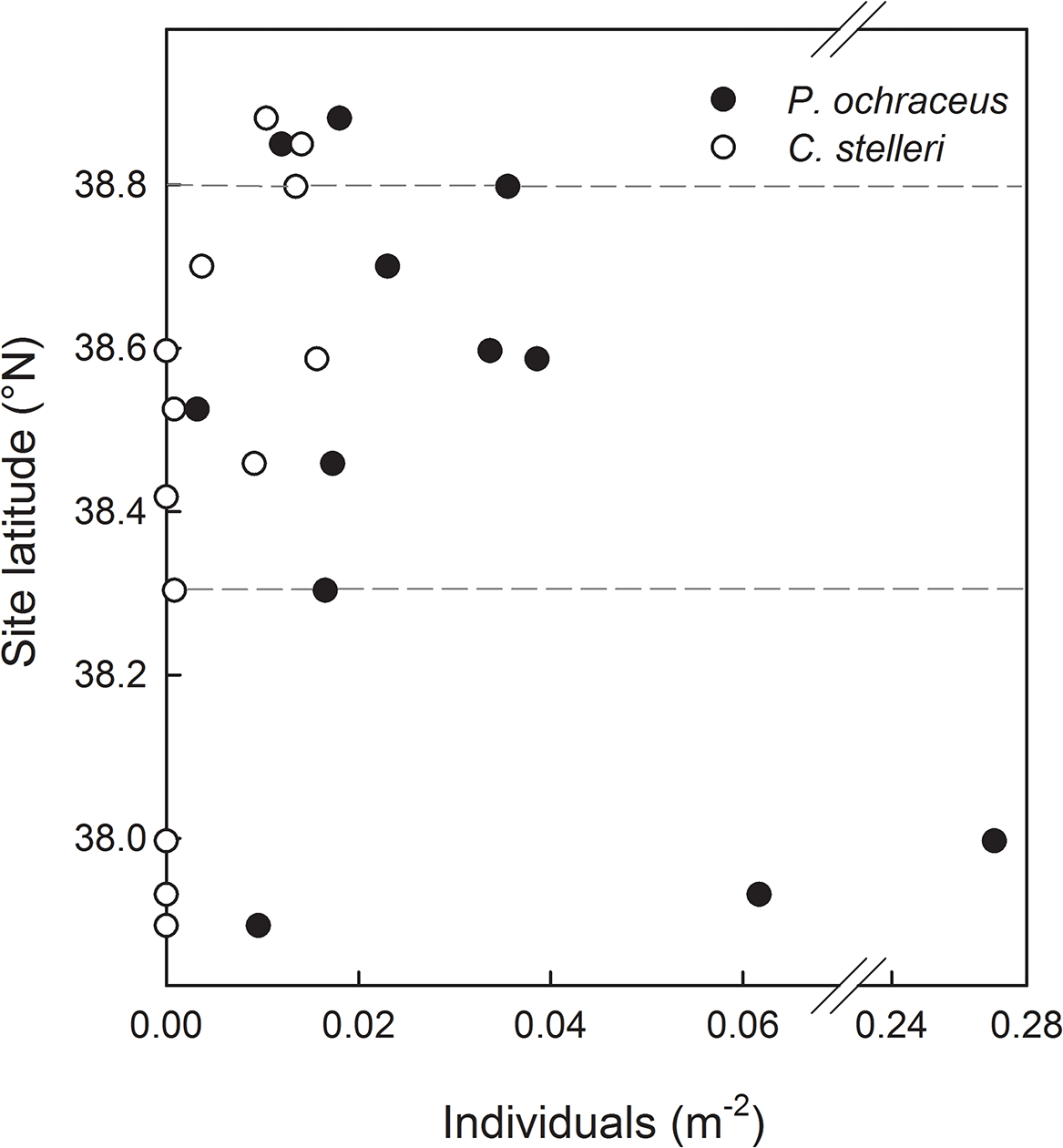
Post-event densities of *P*. *ochraceus* and *C*. *stelleri*. Data are from swath transects, shown by site latitude. Despite many carcasses of these species being observed on shores during the die-off, surveys did not show consistently depressed density within the impact zone (defined by the loss of *S*. *purpuratus* and *Leptasterias* sp. and indicated by dashed lines). See S3 Table for raw counts and area surveyed per site.

Ochre sea stars and gumboot chitons also experienced elevated mortality, with hundreds of dead animals washed up on beaches (Fig 1). However, we found numerous surviving *P*. *ochraceus* and *C*. *stelleri* within the region where *S*. *purpuratus* and *Leptasterias* sp. were lost (Fig 7). *Pisaster ochraceus* density across five sites within the impact zone where pre-event monitoring data were available (sites 7, 8, 10, 14, 19) showed no significant change between 2010 and 2012 (t_4_ = 0.15, *P* = 0.45), and average density decreased only slightly, from 0.14 to 0.12 individuals per m^2^. Swath transects, which covered large portions of each site, revealed complex spatial patterns (Fig 7, S3 Table) and no evidence of consistently lower densities inside versus outside the impact zone for either *P*. *ochraceus* or *C*. *stelleri* (*U* = 16.0, *P* = 0.62 for each species). Gumboot chitons were absent at the southernmost three intertidal-zone sites (sites 22–24), but this observation may be due to their scarcity in intertidal habitats south of Point Reyes, although they remain common in subtidal habitats as far south as Monterey Bay [48].

Based on the spatial pattern of abundance in surveys conducted between 2012 and 2014, the sea star *Henricia* sp. may also have been affected (S4 Table). Following the event, we found multiple individuals at sites to the north and south of the impact zone and only a single individual within the impact zone (S4 Table). However, it is difficult to make firm conclusions concerning the effects of this event on *Henricia* sp. There were no captive *Henricia* sp. at BML when the death of other species was observed in the laboratory, and *Henricia* sp. had not been abundant inside the impact zone, or the entire region, prior to August 2011 (8 individuals were found in all surveys between 2001 and 2010 across 8 sites).

### Biotic and abiotic conditions coincident with mortality

We observed strongly discolored seawater indicating high near-shore plankton concentrations beginning August 24, 2011 and lasting for the next week. Chlorophyll-a data suggest a phytoplankton bloom early in the month, and a second bloom beginning around August 22, which peaked between August 26 and 29, 2011 at approximately 5 ug/L (S1 Fig). During the event, dissolved oxygen remained near saturation (98% DO; J. Moore *pers*. *comm*.) in flow-through seawater tanks at BML that sourced water directly from the adjacent affected site (site 18, Bodega Marine Reserve). Wave heights, salinities, and air and water temperatures were typical for the region over the entire month of August 2011, with air and water temperatures ranging between 10 and 14°C (S1 Fig). Offshore swell heights of 2 to 4 m led to 2 to 5 m waves breaking on the shore, which are also typical of the region (S1 Fig).

## Discussion

In late August and September 2011, intertidal populations of two echinoderms—*S*. *purpuratus* and *Leptasterias* sp.—were decimated over a large, continuous region. We found only ten surviving intertidal purple urchins out of a prior regional population we estimate at many millions of individuals. For both intertidal *S*. *purpuratus* and *Leptasterias* sp., the overall mortality rate was therefore >99.99% over 100 km of coastline. At subtidal locations, mortality of *S*. *purpuratus* was severe but incomplete. A third echinoderm, *Henricia* sp., also may have suffered elevated mortality, but firm conclusions are not possible given its rarity prior to the event.

Two other species, the echinoderm *P*. *ochraceus* and the mollusc *C*. *stelleri*, suffered mortality in the same region and at the same time as *S*. *purpuratus* and *Leptasterias* sp., as evinced by dead animals on beaches (e.g., Fig 1). However, the measured effect on *P*. *ochraceus* and *C*. *stelleri* population sizes was non-significant, possibly influenced by high pre-event variation in regional population sizes. Beach stranding observations may have been biased toward these species because of their heavy, durable carcasses. Such bias was evident for other species; during diver surveys, individuals who directly observed dead animals estimated 25% mortality in red abalone *H*. *rufescens* and 50 to 90% mortality of shallow-living red urchin *M*. *franciscanus*, but only the heavy-bodied *H*. *rufescens* was found on beaches (LRB *pers*. *obs*., [75]).

The geographic scale, rapidity, and taxonomic scope of this mass mortality event are notable, as is the high mortality rate for purple urchins and six-armed sea stars. Although localized mass mortalities are not uncommon for *S*. *purpuratus* [40, 76, 77], no previously documented mortality event has been so severe over such a large region (reviewed in [40, 78]; see also Table 1). Even in comparison with high-severity events, an apparently uniform lack of survivors in certain taxa (i.e., *Leptasterias* in this study) following an event is uncommon. Loss of *Heliaster kubiniji* from the Gulf of California left remnant densities of 0.05 per m^2^ in some locations [79], *Zostera marina* survived the 1930’s die-off along the Atlantic seaboard in low-salinity refugia [80, 81], and the impacts of an unknown pathogen on long-spined sea urchins in the Caribbean were spatially variable [18]. To date, the current progression of sea star wasting disease on the west coast of North America has also left numerous survivors in many locations [82]. The event documented here was also unusual in its sudden onset and brief time-course, a pattern that is rare in marine systems except in cases of discrete physical disturbance like hurricanes. Large-scale marine mortalities typically take several months or years to progress. For example, a green urchin (*Strongylocentrotus droebachiensis*) die-off in Nova Scotia, eventually attributed to an amoeba [83], began in 1980, but did not reach peak mortality until 1984 [84, 85]; decline of black abalone (*Haliotis cracherodii*) in California manifested over decades [86, 87]; and dogwhelk mortality associated with a 1988 algal bloom on the Norwegian coast occurred over several months [88]. The unique attributes of the mass mortality we describe here therefore provide a rare opportunity for subsequent investigations into response and recovery dynamics following a strong and discrete perturbation.

### Potential causes: Evidence and implications

As is the case for many documented mass-mortality events, we cannot unambiguously ascribe the current die-off to a particular cause or set of causes. However, the combination of synchronous and spatially consistent mortality in multiple species, juxtaposed with zero detectable effect in adjacent regions, is consistent with a single, geographically constrained agent. Conceivable causes of the patterns we observed include five, potentially nonexclusive, processes: (1) physical disturbance, (2) mass migration of individuals to areas outside the sampling domain, (3) dysoxia or anoxia, (4) a disease outbreak, and (5) toxicity resulting from a harmful algal bloom (HAB). Of these, toxicity from a HAB is most likely. We detected no evidence of unusual physical disturbance in oceanographic or weather conditions, including temperature, salinity, wind, or wave action during the period of increased mortality. A scenario in which multiple, largely sedentary and slow-moving taxa abruptly exit a long-occupied area of suitable habitat appears implausible on principle, and even less likely given that adult purple urchins often outgrow their burrow entrances and become trapped within them ([89]; LJJ, *pers*. *obs*.). Wave exposure during the event was sufficient to enable energetic wave breaking on rocky shores [90–92], which results in regular, rapid injections of oxygen into nearshore habitats, making widespread hypoxia unlikely. Mortality of laboratory-held urchins and *Leptasterias* sp. also occurred at measured, near-saturation oxygen levels, further indicating an alternative cause. A disease outbreak is plausible, but in contrast to the usual progression of diseases, the event we describe affected multiple phyla and exhibited no indication of a propagating pathogenic front. Moreover, results suggest that mortality occurred acutely across the full range of the impacted zone, and exhibited sharp lines of demarcation between it and adjacent areas—atypical traits for a transmissible disease.

The most likely cause of this mortality event therefore appears to be a toxin produced by phytoplankton that bloomed concurrently with the die-off. Mortality of echinoderms and gastropods following HABs has been documented, but less commonly than effects on fishes and bivalves [93]. The toxicity hypothesis is supported by genetic analyses that show surviving *H*. *rufescens* had disproportionately up-regulated genes that respond to yessotoxin [75]. Yessotoxins are produced by the dinoflagellate genus *Gonyaulax* [94, 95], a species of which (*G*. *membranacea*) was isolated from water samples as the most abundant species during initial phases of the bloom (C. O’Kelly, *pers*. *comm*.). Although yessotoxin-producing species have been identified as an emerging concern in northeast Pacific ecosystems [46], yessotoxins have not previously been known as lethal. That said, the dynamics of microalgal toxicity are still poorly understood, even for better-known HAB species (e.g., [96]), and the possibility remains that unidentified species and/or toxin(s) were responsible. Dinoflagellate cysts can persist for years in sediment and may bloom again under favorable conditions [97], so future events in the region are possible, even as ecological impacts from the current mass mortality progress.

### Community repercussions and recovery potential

The range of mortality rates observed across the five species surveyed suggests the potential for substantial variation in population impacts, species-specific recovery, and community response. In particular, the high mortality in *S*. *purpuratus* (>99.99% intertidally, ~97% subtidally) and *Leptasterias* sp. (~100%) within the affected region increases the probability of prolonged recovery failure in these taxa because recolonization is likely to rely heavily on immigration from sources outside the region (e.g., [19, 20, 98]). However, these two species’ differing life histories may also shape distinct recovery trajectories. Purple urchins produce many larvae that spend several weeks feeding in the plankton [99]. In contrast, six-armed sea stars produce few crawl-away juveniles. Such differences in fecundity and pelagic duration tend to be associated with distinct differences in dispersal potential [100–102]. As a consequence, there is a much greater probability that larvae produced outside the impact zone by *S*. *purpuratus* will settle and recruit inside it, compared to offspring produced by *Leptasterias* adults outside the die-off area. The net result is that although both species may mature in as little as one-to-two years [71, 103], allowing breeding populations to re-establish quickly if juveniles settle in high densities, delivery of new individuals to the impact zone is more likely to operate as a limiting step in the recovery of *Leptasterias* sp. than in *S*. *purpuratus*.

Predicting community responses to this mortality event is complicated. One challenge stems from a degree of uncertainty about the species’ ecological roles. For example, the paradigm established for subtidal *S*. *purpuratus* is that they often graze actively in kelp forests [76, 104, 105], such that their removal can induce rapid shifts to a macroalgae-dominated community [106–108]. However, intertidal purple urchins in the study region subsist primarily on abundant drift algae. They rarely move, and are often incapable of mobile foraging after outgrowing the openings to their burrows [53, 89]. The effect of region-wide loss of intertidal *S*. *purpuratus* might therefore manifest primarily at the ecosystem level, via reductions in nutrient cycling associated with algal detritus. Standing algal stock, by contrast, could be affected more by the loss of the predator *Leptasterias* sp., which indirectly affects grazing rates of herbivorous snails [109]. Despite lower mortality rates in *P*. *ochraceus* and *C*. *stelleri*, reductions in the populations of these species could conceivably affect community structure through food web interactions that alter ratios of habitat-forming mussel beds and algae [110–112]. Moreover, in the case of *P*. *ochraceus*, such effects could be exacerbated by further declines due to the recent regional outbreak of sea star wasting disease.

Quantification of the die-off documented here thus provides a relatively unusual example of a spatially consistent and discrete mortality event with severe to modest impacts in a suite of species. Results establish new regional baselines of population abundance and distribution, and underscore the potential for acute biological disturbances to drive potentially long-term effects on population, community, and ecosystem dynamics. Documenting such disturbance events and their manner of recovery will become increasingly important if and when global changes escalate the magnitude and frequency of local to regional-scale environmental perturbations [113].

## Supporting Information

S1 Fig Abiotic conditions in August 2011. Data shown are hourly averages of (A) shoreline salinity, (B) seawater chlorophyll-a, (C) significant wave height (SWH), and (D) air and water temperatures at Bodega Marine Reserve (site 18) before and during observed invertebrate mortality, the onset of which (first observed Aug 28, 2011) is indicated by a dashed vertical line in each plot. SWH data are from NOAA NDBC Station 46013. All other data are from sensors deployed by Bodega Ocean Observing Node; descriptions of sensors, deployments and quality control can be found at: http://bml.ucdavis.edu/boon/datasets.html.
(PDF)

S1 File Supplemental methods information. Description of methods used in literature review to construct Table 1; full description of swath transect methods with details of additional site locations and survey dates, and information about tissue collections.
(PDF)

S1 Table Detail of site locations and surveys. Includes GPS locations of sites, types and dates of surveys, special jurisdictions for each site and details of relevant permits.
(PDF)

S2 Table Counts of *Leptasterias* sp. Table includes counts of individuals found in 0.25 m^2^ quadrats in surveys conducted before (2001 to 2010) and after (2012) the mass mortality event, by site location, with area surveyed.
(PDF)

S3 Table Counts of *P*. *ochraceus* and *C*. *stelleri* in swath transects. Table includes numbers of each species found in 2012 in large swath transects conducted at each site, with survey dates and area of coverage.
(PDF)

S4 Table Counts of *Henricia* sp. Table includes numbers of *Henricia* sp. found in 0.25 m^2^ quadrats between 2012 and 2014, with locations, dates, and area surveyed.
(PDF)

## References

[1] Hughes TP. Catastrophes, phase shifts, and large-scale degradation of a Caribbean coral reef. Science. 1994;265: 1547–1551. 17801530 10.1126/science.265.5178.1547

[2] Mangel M, Levin PS. Regime, phase and paradigm shifts: making community ecology the basic science for fisheries. Philos Trans R Soc Lond B Biol Sci. 2005;360: 95–105. 15713590 10.1098/rstb.2004.1571 PMC1636097

[3] Paine RT, Tegner MJ, Johnson EA. Compounded perturbations yield ecological surprises. Ecosystems. 1998;1: 535–545.

[4] Easterling DR, Meehl GA, Parmesan C, Changnon SA, Karl TR, Mears LO. Climate extremes: Observations, modeling, and impacts. Science. 2000;289: 2068–2074. 11000103 10.1126/science.289.5487.2068

[5] Hansen J, Sato M, Ruedy R. Perception of climate change. Proc Natl Acad Sci U S A. 2012;109: E2415–E2423. 10.1073/pnas.1205276109 22869707 PMC3443154

[6] Woodley J, Chornesky E, Cliffo P, Jackson J, Kaufman L, Knowlton N, et al Hurricane Allen's impact on Jamaican coral reefs. Science. 1981;214: 749–755. 17744383 10.1126/science.214.4522.749

[7] Harvell CD, Kim K, Burkholder J, Colwell R, Epstein PR, Grimes D, et al Emerging marine diseases–climate links and anthropogenic factors. Science. 1999;285: 1505–1510. 10498537 10.1126/science.285.5433.1505

[8] Harvell CD, Mitchell CE, Ward JR, Altizer S, Dobson AP, Ostfeld RS, et al Climate warming and disease risks for terrestrial and marine biota. Science. 2002;296: 2158–2162. 12077394 10.1126/science.1063699

[9] Harley C. Tidal dynamics, topographic orientation, and temperature-mediated mass mortalities on rocky shores. Mar Ecol Prog Ser. 2008;371: 37–46.

[10] Hallegraeff GM. Ocean climate change, phytoplankton community responses, and harmful algal blooms: A formidable predictive challenge. J Phycol. 2010;46: 220–235.

[11] The Science and Philosophy of Edward Flanders Robb Ricketts (1897–1948); Stanford University Archives [Internet]. Available: http://edricketts.stanford.edu/cards.html. Accessed 3 December 2014.

[12] Tegner MJ, Dayton PK. El Niño Effects on Southern California kelp forest communities. Adv Ecol Res. 1987;17: 243–279.

[13] Dayton PK, Tegner MJ. Bottoms beneath troubled waters: benthic impacts of the 1982–1984 El Niño in the temperate zone. Elsevier Oceanography Series. 1990;52: 433–472.

[14] Schimmelmann A, Tegner MJ. Historical oceanographic events reflected in 13C/12C ratio of total organic carbon in laminated Santa Barbara Basin sediment. Global Biogeochem Cycles. 5;1991: 173–188.

[15] Harger BW. A historical overview of kelp in southern California In: Bascom W, editor. The Effects of Waste Disposal on Kelp Communities. Long Beach: Southern California Coastal Water Research Project; 1983 pp. 70–83.

[16] Carlton JT, Vermeij GJ, Lindberg DR, Carlton DA, Dubley E. The first historical extinction of a marine invertebrate in an ocean basin: the demise of the eelgrass limpet *Lottia alveus* . Biol Bull. 1991;180: 72–80. 29303643 10.2307/1542430

[17] Cottam C, Lynch JJ, Nelson AL. Food habits and management of American Sea Brant. J Wildl Manage. 1944;8: 36–56.

[18] Lessios H. Mass mortality of *Diadema antillarum* in the Caribbean: what have we learned? Annu Rev Ecol Syst. 1988;19: 371–393.

[19] Lessios HA. *Diadema antillarum* 10 years after mass mortality: still rare, despite help from a competitor. Proc R Soc Lond B Biol Sci. 1995;259: 331–337.

[20] Karlson RH, Levitan DR. Recruitment limitation in open populations of *Diadema antillarum*: an evaluation. Oecologia. 1990;82: 40–44. 28313135 10.1007/BF00318531

[21] Diaz RJ. Overview of hypoxia around the world. J Environ Qual. 2001;30: 275–281. 11285887 10.2134/jeq2001.302275x

[22] Cerrano C, Bavestrello G. Mass mortalities and extinctions In: Wahl M, editor. Marine Hard Bottom Communities: Patterns, Dynamics, Diversity, and Change. Berlin Heidelberg: Springer-Verlag; 2009 pp. 295–307.

[23] Cerrano C, Totti C, Sponga F, Bavestrello G. Summer disease in *Parazoanthus axinellae* (Schmidt, 1862) (Cnidaria, Zoanthidea). Ital J of Zool. 2006;73: 355–361.

[24] Garrabou J, Coma R, Bensoussan N, Bally M, Chevaldonne P, Cigliano M, et al Mass mortality in Northwestern Mediterranean rocky benthic communities: effects of the 2003 heat wave. Glob Chang Biol. 2009;15: 1090–1103.

[25] Girard D, Clemente S, Toledo-Guedes K, Brito A, Hernández JC. A mass mortality of subtropical intertidal populations of the sea urchin *Paracentrotus lividus*: analysis of potential links with environmental conditions. Mar Ecol. 2012;33: 377–385.

[26] Scheibling R, Feehan C, Lauzon-Guay J. Disease outbreaks associated with recent hurricanes cause mass mortality of sea urchins in Nova Scotia. Mar Ecol Prog Ser. 2010;408: 109–116.

[27] Stokesbury KDE, Harris BP, Marino MC, Nogueira JI. Sea scallop mass mortality in a Marine Protected Area. Mar Ecol Prog Ser. 2007;349: 151–158.

[28] Anthony KRN, Kerswell AP. Coral mortality following extreme low tides and high solar radiation. Mar Biol. 2007;151: 1623–1631.

[29] Dupont JM, Hallock P, Jaap WC. Ecological impacts of the 2005 red tide on artificial reef epibenthic macroinvertebrate and fish communities in the eastern Gulf of Mexico. Mar Ecol Prog Ser. 2010;415: 189–200.

[30] Eakin CM, Morgan JA, Heron SF, Smith TB, Liu G, Alvarez-Filip L, et al Caribbean corals in crisis: record thermal stress, bleaching, and mortality in 2005. PloS ONE. 2010;5: e13969 10.1371/journal.pone.0013969 21125021 PMC2981599

[31] Hernandez-Miranda E, Quinones RA, Aedo G, Diaz-Cabrera E, Cisterna J. The impact of a strong natural hypoxic event on the toadfish *Aphos porosus* in Coliumo Bay, south-central Chile. Rev Biol Mar Oceanogr. 2012;47: 475–487.

[32] Hernandez-Miranda E, Veas R, Labra FA, Salamanca M, Quinones RA. Response of the epibenthic macrofaunal community to a strong upwelling-driven hypoxic event in a shallow bay of the southern Humboldt Current System. Mar Environ Res. 2012;79: 16–28. 10.1016/j.marenvres.2012.04.004 22626877

[33] Huete-Stauffer C, Vielmini I, Palma M, Navone A, Panzalis P, Vezzulli L, et al *Paramuricea clavata* (Anthozoa, Octocorallia) loss in the Marine Protected Area of Tavolara (Sardinia, Italy) due to a mass mortality event. Mar Ecol Evol Persp. 2011;32: 107–116.

[34] Cebrian E, Jesus Uriz M, Garrabou J, Ballesteros E. Sponge mass mortalities in a warming Mediterranean Sea: are cyanobacteria-harboring species worse off? PloS ONE. 2011;6: e20211 10.1371/journal.pone.0020211 21673798 PMC3105983

[35] Stabili L, Cardone F, Alifano P, Tredici SM, Piraino S, Corriero G, et al Epidemic mortality of the sponge *Ircinia variabilis* (Schmidt, 1862) associated to proliferation of a *Vibrio* bacterium. Microb Ecol. 2012;64: 802–813. 22573240 10.1007/s00248-012-0068-0

[36] Di Camillo CG, Bartolucci I, Cerrano C, Bavestrello G. Sponge disease in the Adriatic Sea. Mar Ecol Evol Persp. 2013;34: 62–71.

[37] Micheli F, Saenz-Arroyo A, Greenley A, Vazquez L, Espinoza Montes JA, Rossetto M, et al Evidence that marine reserves enhance resilience to climatic impacts. PLoS ONE. 2012;7: e40832 10.1371/journal.pone.0040832 22855690 PMC3408031

[38] Benítez-Villalobos F, Diaz Martinez J, Martínez-García M. Mass mortality of the sea urchin *Diadema mexicanum* in La Entrega at Bahias de Huatulco, Western Mexico. Coral Reefs. 2009;28: 1017–1017.

[39] Kemp DW, Oakley CA, Thornhill DJ, Newcomb LA, Schmidt GW, Fitt WK. Catastrophic mortality on inshore coral reefs of the Florida Keys due to severe low-temperature stress. Glob Chang Biol. 2011;17: 3468–3477.

[40] Hendler G. Recent mass mortality of *Strongylocentrotus purpuratus* (Echinodermata: Echinoidea) at Malibu and a review of purple sea urchin kills elsewhere in California. Bull So Calif Acad Sci. 2013;112: 19–37.

[41] Försterra G, Häussermann V, Laudien J, Jantzen C, Sellanes J, Muñoz P. Mass die off of the cold-water coral *Desmophyllum dianthus* in the Chilean Patagonian fjord region. Bull Mar Sci. 2014;90: 895–899.

[42] Hewson I, Button JB, Gudenkauf BM, Miner B, Newton AL, Gaydos JK, et al *Densovirus* associated with sea-star wasting disease and mass mortality. Proc Natl Acad Sci U S A: 2014;111: 17278–17283. 10.1073/pnas.1416625111 25404293 PMC4260605

[43] Huyer A. Coastal upwelling in the California Current system. Prog Oceanogr. 1983;12: 259–284.

[44] Largier J, Magnell B, Winant C. Subtidal circulation over the northern California shelf. J Geophys Res Oceans (1978–2012). 1993;98: 18147–18179.

[45] Kudela RM, Seeyave S, Cochlan WP. The role of nutrients in regulation and promotion of harmful algal blooms in upwelling systems. Prog Oceanogr. 2010;85: 122–135.

[46] Lewitus AJ, Horner RA, Caron DA, Garcia-Mendoza E, Hickey BM, Hunter M, et al Harmful algal blooms along the North American west coast region: history, trends, causes, and impacts. Harmful Algae. 2012;19: 133–159.

[47] Gotshall DW, Lea RN, Laurent LL, Hoban TL. Mendocino Power Plant site ecological study annual report; July 1, 1971 to December 31, 1972. Long Beach (CA): California Department of Fish and Game, Marine Resources Division; 1973 Administrative Report 73–4.

[48] Ricketts EF. Between Pacific Tides. 5th ed. Stanford (CA): Stanford University Press; 1985.

[49] Ebert TA, Russell MP. Latitudinal variation in size structure of the west coast purple sea urchin: a correlation with headlands. Limnol Oceanogr. 1988;33: 286–294.

[50] Ebert TA. Demographic patterns of the purple sea urchin *Strongylocentrotus purpuratus* along a latitudinal gradient, 1985–1987. Mar Ecol Prog Ser. 2010;406: 105–120.

[51] Clark HL. Echinoderms from Lower California, with descriptions of new species New York: American Museum of Natural History; 1913.

[52] Duggins DO. Sea urchins and kelp–the effects of short-term changes in urchin diet. Limnol Oceanogr. 1981;26: 391–394.

[53] Lester SE, Gaines SD, Kinlan BP. Reproduction on the edge: Large-scale patterns of individual performance in a marine invertebrate. Ecology. 2007;88: 2229–2239. 17918401 10.1890/06-1784.1

[54] Menge B. Brood or broadcast? The adaptive significance of different reproductive strategies in the two intertidal sea stars *Leptasterias hexactis* and *Pisaster ochraceus* . Mar Biol. 1975;31: 87–100.

[55] Paine RT. Food web complexity and species diversity. Am Nat. 1966;100: 65–75.

[56] Menge BA, Berlow EL, Blanchette CA, Navarrete SA, Yamada SB. The keystone species concept: variation in interaction strength in a rocky intertidal habitat. Ecol Monogr. 1994;64: 249–286.

[57] Foltz DW. Hybridization frequency is negatively correlated with divergence time of mitochondrial DNA haplotypes in a sea star (*Leptasterias* spp.) species complex. Evolution. 1997;51: 283–288. 28568776 10.1111/j.1558-5646.1997.tb02410.x

[58] Flowers J, Foltz DW. Reconciling molecular systematics and traditional taxonomy in a species-rich clade of sea stars (*Leptasterias* subgenus *Hexasterias*). Mar Biol. 2001;139: 475–483.

[59] Foltz DW, Nguyen AT, Kiger JR, Mah CL. Pleistocene speciation of sister taxa in a North Pacific clade of brooding sea stars (*Leptasterias*). Mar Biol. 2008;154: 593–602.

[60] Menge JL, Menge BA. Role of resource allocation, aggression and spatial heterogeneity in coexistence of two competing intertidal starfish. Ecol Monogr. 1974;44: 189–209.

[61] Phillips D. Life-history features of the marine intertidal limpet *Notoacmea scutum* (Gastropoda) in central California. Mar Biol. 1981;64: 95–103.

[62] Thomas LA, Hermans CO. Adhesive interactions between the tube feet of a starfish, *Leptasterias hexactis*, and substrata. Biol Bull. 1985;169: 675–688.

[63] Lord JP. Larval development, metamorphosis and early growth of the gumboot chiton *Cryptochiton stelleri* (Middendorff, 1847) (Polyplacophora: Mopaliidae) on the Oregon coast. J Molluscan Stud. 2011;77: 182–188.

[64] Fewkes JW. On Excavations Made in Rocks by Sea Urchins. Am Nat. 1890;24: 1–21.

[65] Denny M, Shibata M. Consequences of surf-zone turbulence for settlement and external fertilization. Am Nat. 1989;134: 859–889.

[66] Cameron R, Schroeter S. Sea urchin recruitment: effect of substrate selection on juvenile distribution. Mar Ecol Prog Ser. 1980;2: 243–247.

[67] Kalvass P, Taniguchi I, Buttolph P, DeMartini J. Relative abundance and size composition of red sea urchin, *Strongylocentrotus franciscanus*, populations along the Mendocino and Sonoma County coasts, 1989. Fort Bragg (CA): California Department of Fish and Game, Marine Resources Division; 1991 Marine Resources Administrative Report, 91–3.

[68] Pennington JT. The ecology of fertilization of echinoid eggs: the consequences of sperm dilution, adult aggregation, and synchronous spawning. Biol Bull. 1985;169: 417–430. 29314924 10.2307/1541492

[69] Levitan DR. Influence of body size and population density on fertilization success and reproductive output in a free-spawning invertebrate. Biol Bull. 1991;181: 261–268. 29304646 10.2307/1542097

[70] Mead KS, Denny MW. The effects of hydrodynamic shear stress on fertilization and early development of the purple sea urchin *Strongylocentrotus purpuratus* . Biol Bull. 1995;188: 46–56. 7696387 10.2307/1542066

[71] Gonor JJ. Gonad growth in the sea urchin, *Strongylocentrotus purpuratus* (Stimpson) (echinodermata: Echinoidea) and the assumptions of gonad index methods. J Exp Mar Bio Ecol. 1972;10: 89–103.

[72] Eernisse DJ, Strathmann MF, Strathmann RR. *Henricia pumila* sp. nov.: a brooding seastar (Asteroidea) from the coastal northeastern Pacific. Zootaxa. 2010;2329: 22–36. 10.1109/IEMBS.2010.5627464 21096802 PMC3764051

[73] Mah CL, Blake DB. Global Diversity and Phylogeny of the Asteroidea (Echinodermata). PLoS ONE. 2012;7: e35644 10.1371/journal.pone.0035644 22563389 PMC3338738

[74] Rogers-Bennett L, Kudela R, Nielsen K, Paquin A, O’Kelly C, Langlois G, et al Dinoflagellate bloom coincides with marine invertebrate mortalities in northern California. Harfmful Algae News. 2012;46: 10–11.

[75] De Wit P, Rogers-Bennett L, Kudela RM, Palumbi SR. Forensic genomics as a novel tool for identifying the causes of mass mortality events. Nat Commun. 2014 Apr 16. 10.1038/ncomms4652 24736548

[76] Pearse JS, Hines AH. Expansion of a central California kelp forest following the mass mortality of sea urchins. Mar Biol. 1979;51: 83–91.

[77] Uthicke S, Schaffelke B, Byrne M. A boom-bust phylum? Ecological and evolutionary consequences of density variations in echinoderms. Ecol Monogr. 2009;79: 3–24.

[78] Rogers-Bennett L. *Strongylocentrotus franciscanus* and *Strongylocentrotus purpuratus* In: Lawrence JM, editor. Sea urchins: biology and ecology. Oxford: Elsevier; 2013 pp. 413–436.

[79] Dungan ML, Miller TE, Thomson DA. Catastrophic decline of a top carnivore in the Gulf of California rocky intertidal zone. Science. 1982;216: 989–991. 17809070 10.1126/science.216.4549.989

[80] Rasmussen E. The wasting disease of eelgrass (*Zostera marina*) and its effects on environmental factors and fauna In: McRoy CP, Helfferich C, editors. Seagrass Ecosystems, A Scientific Perspective. New York: Marcel Dekker; 1977 pp. 1–51.

[81] Short F, Mathieson A, Nelson J. Recurrence of the eelgrass wasting disease at the border of New Hampshire and Maine, USA. Mar Ecol Prog Ser. 1986;29: 89–92.

[82] Stokstad E. Death of the Stars. Science. 2014;344: 464–467. 10.1126/science.344.6183.464 24786059

[83] Jones G, Scheibling R. *Paramoeba* spp.(Amoebida, Paramoebidae) as the possible causative agent of sea urchin mass mortality in Nova Scotia. Journal Parasitol. 1985;71: 559–565. 4057000

[84] Miller RJ, Colodey AG. Widespread mass mortalities of the green sea urchin in Nova Scotia, Canada. Mar Biol. 1983;73: 263–267.

[85] Scheibling R. Increased macroalgal abundance following mass mortalities of sea urchins (*Strongylocentrotus droebachiensis*) along the Atlantic coast of Nova Scotia. Oecologia. 1986;68: 186–198. 28310126 10.1007/BF00384786

[86] Altstatt JM, Ambrose RF, Engle JM, Haaker PL, Lafferty KD, Raimondi PT. Recent declines of black abalone *Haliotis cracherodii* on the mainland coast of central California. Mar Ecol Prog Ser. 1996;142: 185–192.

[87] Raimondi PT, Wilson CM, Ambrose RF, Engle JM, Minchinton TE. Continued declines of black abalone along the coast of California: Are mass mortalities related to El Niño events? Mar Ecol Prog Ser. 2002;242: 143–152.

[88] Robertson A. Effects of a toxic bloom of *Chrysochromulina polylepis* on the common dog-whelk, *Nucella lapillus* on the Swedish west coast. J Mar Biol Assoc U. K. 1991;71: 569–578.

[89] Grupe BM. Purple sea urchins (*Strongylocentrotus purpuratus*) in and out of pits: the effects of microhabitat on population structure, morphology, growth, and mortality [MS thesis] Eugene (OR): University of Oregon; 2006.

[90] Gaylord B. Detailing agents of physical disturbance: wave-induced velocities and accelerations on a rocky shore. J Exp Mar Bio Ecol. 1999;239: 85–124.

[91] Gaylord B. Biological implications of surf-zone flow complexity. Limnol Oceanogr. 2000;45: 174–188.

[92] Gaylord B, Hodin J, Ferner MC. Turbulent shear spurs settlement in larval sea urchins. Proc Natl Acad Sci U S A. 2013;110: 6901–6906. 10.1073/pnas.1220680110 23572585 PMC3637773

[93] Landsberg JH. The effects of harmful algal blooms on aquatic organisms. Rev Fish Sci. 2002;10: 113–390.

[94] Rhodes L, McNabb P, De Salas M, Briggs L, Beuzenberg V, Gladstone M. Yessotoxin production by *Gonyaulax spinifera* . Harmful Algae. 2006;5: 148–155.

[95] Howard MD, Smith GJ, Kudela RM. Phylogenetic relationships of yessotoxin-producing dinoflagellates, based on the large subunit and internal transcribed spacer ribosomal DNA domains. Appl Environ Microb. 2009;75: 54–63. 10.1128/AEM.00818-08 PMC2612195 19011074

[96] Gobler CJ, Sunda WG. Ecosystem disruptive algal blooms of the brown tide species, *Aureococcus anophagefferens* and *Aureoumbra lagunensis* . Harmful Algae. 2012;14: 36–45.

[97] Hallegraeff GM. A review of harmful algal blooms and their apparent global increase. Phycologia. 1993;32: 79–99.

[98] Knowlton N. Multiple “stable” states and the conservation of marine ecosystems. Progr Oceanogr. 2004;60: 387–396.

[99] Strathmann R. Length of pelagic period in echinoderms with feeding larvae from the Northeast Pacific. J Exp Mar Bio Ecol. 1978;34: 23–27.

[100] Dawson MN, Hays CG, Grosberg RK, Raimondi PT. Dispersal potential and population genetic structure in the marine intertidal of the eastern North Pacific. Ecol Monogr. 2014;84: 435–456.

[101] Dawson MN. Biogeography and complex traits: dispersal syndromes, in the sea. Front Biogeogr. 2014;6: 11–15.

[102] Riginos C, Douglas KE, Jin Y, Shanahan DF, Treml EA. Effects of geography and life history traits on genetic differentiation in benthic marine fishes. Ecography. 2011;34: 566–575.

[103] Pearse JS, Pearse VB, Davis KK. Photoperiodic regulation of gametogenesis and growth in the sea urchin *Strongylocentrotus purpuratus* . J Exper Zool. 1986;237: 107–118.

[104] Tegner MJ, Dayton PK. Sea urchins El Niños and the long-term stability of southern California kelp forest communities. Mar Ecol Prog Ser. 1991;77: 49–63.

[105] Ebeling AW, Laur DR, Rowley RJ. Severe storm disturbances and reversal of community structure in a southern California kelp forest. Mar Biol. 1985;84: 287–294.

[106] Paine RT, Vadas RL. The effects of grazing by sea urchins, *Strongylocentrotus* spp., on benthic algal populations. Limnol Oceanogr. 1969;14: 710–719.

[107] Leighton D. Grazing activities of benthic invertebrates in southern California kelp beds. Nova Hedwigia. 1971;32: 421–453.

[108] Harrold C, Reed DC. Food availability, sea urchin grazing, and kelp forest community structure. Ecology. 1985;66: 1160–1169.

[109] Gravem SA. Linking antipredator behavior of prey to intertidal zonation and community structure in rocky tidepools [dissertation]. Davis (CA): University of California Davis; 2015.

[110] Paine R. Intertidal community structure. Oecologia. 1974;15: 93–120. 28308255 10.1007/BF00345739

[111] Paine RT. Trophic control of production in a rocky intertidal community. Science. 2002;296: 736–739. 11976455 10.1126/science.1069811

[112] Suchanek TH. The *Mytilus californianus* community: studies on the composition, structure, organization and dynamics of a mussel bed [dissertation]. Seattle (WA): University of Washington; 1979.

[113] Fey SB, Siepielski AM, Nusslé S, Cervantes-Yoshida K, Hwan JL, Huber ER, et al Recent shifts in the occurrence, cause, and magnitude of animal mass mortality events. Proc Natl Acad Sci U S A: 2015;112: 1083–1088. 10.1073/pnas.1414894112 25583498 PMC4313809

