# Correction: Patterns of Mass Mortality among Rocky Shore Invertebrates across 100 km of Northeastern Pacific Coastline

**DOI:** 10.1371/journal.pone.0131969

**Published:** 2015-06-25

**Authors:** The PLOS ONE Staff

There are a number of errors in Table 1. Please see the corrected Table 1 here. The publisher apologizes for these errors.

**Table 1:** Mass mortality events of benthic marine species occurring since 2000.

Year(s), Location	Affected organisms	Suspected cause(s)	Mortality range	Spatial extent (km^2^)^a^ ^,^ ^b^	Spatial pattern reported	References
2001–2003, Ligurian coast, N Mediterranean	Zoanthid (*Parazoanthus axinellae*)	Disease, High water temperature	~ 90%	0.0001	ND	[23]
2003, N Mediterranean	Gorgonians, sponges, bryozoans, bivalves; (multiple species)	High water temperature	5–80% *	1500	patchy	[24]
2003, Canary Islands, SE Atlantic	Sea urchin (*Paracentrotus lividus*)	Disease, high water temperature	0–95%	50	patchy	[25]
2003, 2009, Nova Scotia, NW Atlantic	Sea urchin (*Strongylocentrotus droebachiensis)*	Disease, hurricanes	0–100%	3	patchy	[26]
2004–2005, Cape Cod, NW Atlantic	Sea scallop (*Placopecten magellanicus*)	Unknown	35%	4000	ND	[27]
2005, Great BarrierReef, Coral Sea	Corals (multiple species)	Solar radiation, low tide exposure	10–40% *	10	patchy	[28]
2005, Florida, E Gulf of Mexico	Fishes, sponges (multiple species) Coral (*Cladocora arbuscula*)	Algal bloom, hypoxia	Sponges: 6–7%; other taxa: ND	10	ND	[29]
2005–2007, Caribbean Sea	Corals (multiple species)	High water temperature, disease	0–70%	2 x 10^6^	patchy	[30]
2008, Coliumo Bay, Chile, SE Pacific	Crabs, fishes (multiple species per taxon)	Hypoxia	~90%	5	ND	[31, 32]
2008, Sardinia, N Mediterranean	Octocoral (*Paramuricea clavata*)	Disease, high water temperature	0–100% *	2	ND	[33]
2008, 2009, N Mediterranean	Sponges (*Ircinia* spp.)	Disease, high water temperature	0–95% *	700	patchy	[34–36]
2009, Isla Natividad, Mexico, NE Pacific	Pink abalone (*Haliotis corrugata)*	Hypoxia	41%	10	ND	[37]
2009, Bahia de Huatulco, NE Pacific	Sea urchin (*Diadema mexicanum*)	ND	100%	0.001	ND	[38]
2010, Florida Keys, Straits of Florida	Corals (multiple species)	Low water temperature	17–100%	0.01	ND	[39]
2010, 2011, Malibu, California, NE Pacific	Sea urchin (*Strongylocentrotus purpuratus*)	Low salinity, sediment	0–99%	0.01	patchy	[40]
**2011, Sonoma county, California, NE Pacific**	**Sea urchin (*S*. *purpuratus*), sea star (*Leptasterias* sp.)**	**Harmful algal bloom toxicity**	**>99.99%**	**100**	**continuous**	**this study**
2012, Comau Fjord, Chile, SE Pacific	Coral (*Desmophyllum dianthus*)	Methane and/or sulfide seeps, hypoxia	50–99%	8.4	ND	[41]
2013–present, West coast of N America, NE Pacific	Sea stars (multiple species)	Wasting disease	0–70%	5000	patchy	[42]

Note that the references provided represent to our knowledge the original report(s) describing events in wild populations, and do not include subsequent follow-up publications focused on the same events. Events are summarized from a review of 897 articles; see S1 File for a full description of the literature review methods.

*Denotes mortality of colonial species reported as a percentage of affected colonies with partial necrosis, rather than absolute mortality.

ND, no data.

^a^Where not stated explicitly, we estimated spatial extent of study regions from maps or text descriptions.

^b^Note that most published studies do not include the spatial boundaries of mortality (i.e., the geographic locations past which no mortality was observed). When this information was absent, we report here the spatial extent of the study region.
