# Supplementary material for: Patterns of Mass Mortality among Rocky Shore Invertebrates across 100 km of Northeastern Pacific Coastline
Source: PLoS One. 2015 Jun 3;10(6):e0126280. doi: 10.1371/journal.pone.0126280 (PMC4454560; doi:10.1371/journal.pone.0126280)
Supplement: S1 Fig — Data shown are hourly averages of (A) shoreline salinity, (B) seawater chlorophyll-a, (C) significant wave height (SWH), and (D) air and water temperatures at Bodega Marine Reserve (site 18) before and during observed invertebrate mortality, the onset of which (first observed Aug 28, 2011) is indicated by a dashed vertical line in each plot. SWH data are from NOAA NDBC Station 46013. All other data are from sensors deployed by Bodega Ocean Observing Node; descriptions of sensors, deployments and quality control can be found at: http://bml.ucdavis.edu/boon/datasets.html. (PDF) [file pone.0126280.s001.pdf]

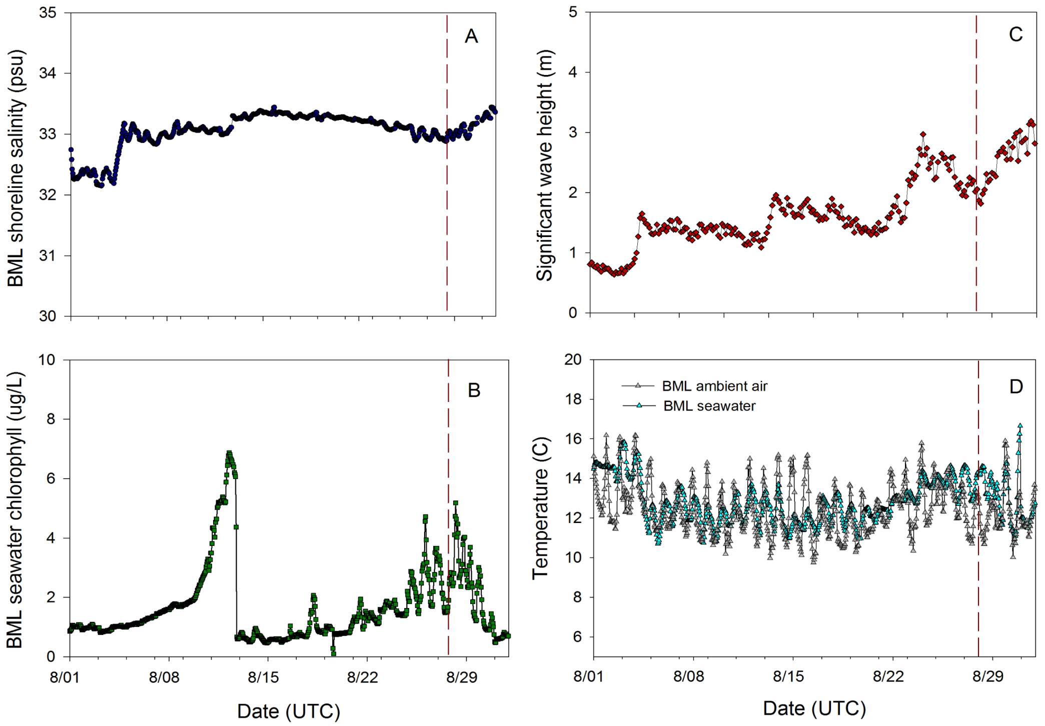

S1 Figure. Abiotic conditions in August 2011. Data shown are hourly averages of (A) shoreline salinity, (B) seawater chlorophyll *a*, (C) significant wave height (SWH), and (D) air and water temperatures at Bodega Marine Reserve (site 18) before and during observed invertebrate mortality, the onset of which (first observed Aug 28, 2011) is indicated by a dashed vertical line in each plot. SWH data are from NOAA NDBC Station 46013. All other data are from sensors deployed by Bodega Ocean Observing Node; descriptions of sensors, deployments and quality control can be found at: http://bml.ucdavis.edu/boon/datasets.html.
