# Supplementary material for: Patterns of Mass Mortality among Rocky Shore Invertebrates across 100 km of Northeastern Pacific Coastline
Source: PLoS One. 2015 Jun 3;10(6):e0126280. doi: 10.1371/journal.pone.0126280 (PMC4454560; doi:10.1371/journal.pone.0126280)
Supplement: S1 File — Description of methods used in literature review to construct Table 1; full description of swath transect methods with details of additional site locations and survey dates, and information about tissue collections. (PDF) [file pone.0126280.s002.pdf]

S1 File. Supporting information describing methods used for Table 1, swath transect surveys and tissue collection.

Benthic mass mortality literature search: Table 1

To compile information (Table 1) on mass mortality events in benthic marine systems that occurred between January 1, 2000 and October, 2014, we conducted a literature search using the electronic databases Web of Science, Scopus, and Google Scholar, last updated on Nov 10, 2014. We included the following topics in single-topic searches: "mass mortality," "mass mortalities," "benthic mortality", "extreme mortality," "catastrophic mortality," "die-off" and "die off." We further refined results to the topic "marine," and the disciplines of ecology, zoology, freshwater and marine biology, oceanography, biodiversity conservation, fisheries, environmental sciences and interdisciplinary sciences, to avoid articles dealing with events in the geologic record and irrelevant disciplines. This approach yielded a total of 897 citations after duplicates were removed. We did all further refinement of the search protocol by reading the abstracts or full articles (691 abstracts, 206 full articles) for each of these papers, to avoid inadvertently excluding relevant studies. Through this effort, we excluded any research articles relating to: pelagic species, marine mammals, captive species, aquaculture, prehistoric events, reviews or meta-analyses unless they included new events, modeling studies without actual mortality events, predator or fishing-related mortality, and events occurring prior to the year 2000 since they had been reviewed elsewhere. We further limited our results to first reports or event descriptions, rather than details of recovery processes or subsequent studies of disease agents. We avoided descriptions of gradual, multi-year population shifts and extirpations of invasive species, because both types of events are driven by processes distinct from the focus of our study. Finally, because repeated instances of similar events in the same location would bias towards particular spatial patterns, we included only the first reports of regularly occurring seasonal events (e.g., seasonal hypoxia). Emerging from these criteria were the 17 events reported in Table 1 along with the one we describe here.

Swath transects: detailed methods, expanded list of sites and survey dates

To provide more thorough estimates of site-specific density for the large, conspicuous *P. ochraceus* and *C. stelleri*, we conducted timed, GPS-tracked, 2 m wide swath transects at each site. Transects ran from the most shoreward to the most seaward suitable habitat for the target species, at approximately 10 m intervals along shore, with auto-recorded trackpoints every 6 seconds. We focused search time at the lowest shore elevations when the tide was lowest, and continued transect searches as long as tidal heights permitted access on each sampling date. To reduce error (commonly +/– 3 m for civilian GPS), we smoothed tracks by averaging across windows of two consecutive trackpoints, and removed outlying trackpoints that led to a Euclidean distance ≥ 8 meters, since these were likely due to temporary drop-outs in satellite signal. We calculated total transect search area by multiplying the adjusted transect length by swath width. We estimated total transect search time using field notes, GPS waypoints and trackpoints, removing 6-second intervals where < 0.05 m were traveled (suggesting this was time during which notes were being written or other data were being collected).

Tissue collection

Where individuals were found, we collected small tissue samples of up to 40 individuals per site of each species of interest (*S. purpuratus, P. ochraceus, C. stelleri, Leptasterias* sp., *Henricia* sp.). We used the least-damaging methods possible, taking spine tissue for urchins, tube feet for sea stars and small pieces of the mantle for chitons. Samples were preserved in 95% ethanol and housed at the University of California Merced, and will be used for genetic analyses by members of our group.
