# Supplementary material for: Patterns of Mass Mortality among Rocky Shore Invertebrates across 100 km of Northeastern Pacific Coastline
Source: PLoS One. 2015 Jun 3;10(6):e0126280. doi: 10.1371/journal.pone.0126280 (PMC4454560; doi:10.1371/journal.pone.0126280)
Supplement: S1 Table — Includes GPS locations of sites, types and dates of surveys, special jurisdictions for each site and details of relevant permits. (PDF) [file pone.0126280.s003.pdf]

S1 Table. Detail of site locations, types and dates of surveys, special jurisdictions and relevant permits.

Site #	Latitude (°N)	Longitude (°W)	Site Name	Research use
1	39.28122	123.80381	Van Damme State Park [a]	B
2	38.91504	123.71107	Arena Cove	B, D
3	38.88121	123.67773	Moat Creek	B, D, F
4	38.84939	123.64752	Iversen Point [b]	B, D, F
5	38.80187	123.58371	Anchor Bay	C
6	38.79659	123.57196	Serenisea	B, C, D
7	38.74103	123.50823	Del Mar Landing [b]	C, D, F
8	38.70013	123.44271	Sea Ranch	A, C, D, F
9	38.59687	123.35058	Fisk Mill Cove [a]	C, D, F
10	38.58653	123.34218	Phillips Gulch [a]	A, B, C, D, F
11	38.56412	123.33283	Salt Point [a,b]	E
12	38.55436	123.30727	Ocean Cove	E
13	38.53065	123.27657	Timber Cove	E
14	38.52501	123.26846	Windermere Point	A, B, C, D, F
15	38.51063	123.24713	Fort Ross [b]	E
16	38.45864	123.14609	Twin Coves [a]	A, B, C, D
17	38.41784	123.10772	Shell Beach [a]	C, D
18	38.31844	123.07403	Bodega Marine Reserve [b,c]	A, B, C, D, F
19	38.30336	123.05275	Bodega Head [a]	C, D, F
20	38.24049	122.99535	Tomales Point [d]	C
21	38.18232	122.96551	McClure's Beach [d]	C, D
22	37.99679	122.97919	Lifeboat House [d]	C, D
23	37.93102	122.74950	Palomarin [d]	B, D
24	37.89309	122.70677	Duxbury Reef [b]	B, D, F

Research uses are for the following surveys (A) Initial observations of mortality in previously abundant *S. purpuratus* populations visited before the event (May–July 2011) and after (Aug–Sep 2011); (B) Initial urchin density surveys conducted Oct–Dec 2011; (C) Fine-grained urchin survivorship surveys: Dec 2011–Dec 2012; (D) Post-event multi-species surveys: Dec 2011–Dec 2013; (E) Subtidal surveys: Aug and Sept 2005–2012 and (F) Pre-event intertidal monitoring surveys, with the last surveys conducted at all sites before the event May–Aug 2010, except Duxbury Reef, last surveyed in Feb 2008. Additional prior surveys were conducted at Sea Ranch in 2001 and 2005, and at Bodega Marine Reserve in 2001, 2003, and 2004.

Bedrock types by site number: 1–8, consolidated mudstone; 9–15, consolidated sandstone and conglomerate; 16 and 17, graywacke and Franciscan mélange; 18–21, granite and diorite; 22, siltstone and mudstone; 23, mudstone, sandstone and soft shale; 24, soft shale.

[a] Locations are within California State Parks; annual permits issued to LJJ by California Department of Parks and Recreation.

[b] Protected as a Marine Protected Area per California Marine Life Protection Act of 1999 (California Department of Fish and Wildlife permits # SC-11596 LJJ, SC-11794 LMS, SC-630 MND).

[c] Location is managed by the University of California Natural Reserve System; permit #26493 to LJJ.

[d] Locations are within Point Reyes National Seashore.
