# Supplementary material for: Patterns of Mass Mortality among Rocky Shore Invertebrates across 100 km of Northeastern Pacific Coastline
Source: PLoS One. 2015 Jun 3;10(6):e0126280. doi: 10.1371/journal.pone.0126280 (PMC4454560; doi:10.1371/journal.pone.0126280)
Supplement: S2 Table — Table includes counts of individuals found in 0.25 m2 quadrats in surveys conducted before (2001 to 2010) and after (2012) the mass mortality event, by site location, with area surveyed. (PDF) [file pone.0126280.s004.pdf]

S2 Table. Counts of *Leptasterias* sp. found in pre-2011 monitoring surveys and 2012 post-event surveys at many of the same sites, in 0.25 m^2 quadrats placed randomly in mid-to-low intertidal zones. All zeros correspond to post-event samples within the die-off zone.

Site Name	Site Latitude	Site Number[a]	Survey year	Count	Area surveyed (m^2)
Point Arena	38.943	NA	2010	12	3
Stornetta	38.938	NA	2004	12	5.5
Stornetta	38.938	NA	2007	10	3
Arena Cove	38.915	2	2012	7	13.25
Moat Creek	38.881	3	2010	10	4
Moat Creek	38.881	3	2012	20	25
Iversen/Saunders Reef	38.850	4	2010	6	6
Iversen/Saunders Reef	38.850	4	2012	2	40.25
Del Mar Landing	38.741	7	2010	20	5.5
Del Mar Landing	38.741	7	2012	0	32
Sea Ranch	38.730	8	2001	28	5.5
Sea Ranch	38.730	8	2005	16	5.5
Sea Ranch	38.730	8	2010	7	3
Sea Ranch	38.730	8	2012	0	34
Phillips Gulch	38.586	9	2010	9	3
Phillips Gulch	38.586	9	2012	0	32
Gerstle Cove	38.566	11	2010	8	3
Windermere Point	38.524	14	2010	4	5.5
Windermere Point	38.524	14	2012	0	32
Bodega Marine Reserve	38.318	18	2001	124	5.5
Bodega Marine Reserve	38.318	18	2003	65	5.5
Bodega Marine Reserve	38.318	18	2004	31	3
Bodega Marine Reserve	38.318	18	2010	17	3
Bodega Marine Reserve	38.318	18	2012	0	26
Bodega Head	38.303	19	2010	21	5.5
Bodega Head	38.303	19	2012	0	37
Santa Maria Creek	38.012	NA	2002	10	5.5
Chimney Rock	37.994	NA	2010	6	3
Bolinas Point	37.905	NA	2002	4	5.5
Bolinas Point	37.905	NA	2005	7	5.5
Bolinas Point	37.905	NA	2008	5	5.5
Bolinas Point	37.903	NA	2005	2	7.25
Palomarin	37.931	23	2012	1	35.5
Duxbury Reef	37.893	24	2008	1	3
Duxbury Reef	37.893	24	2012	16	27.25

[a]Site numbers are given for locations included in the 2012 post-event surveys.
