# Supplementary material for: Patterns of Mass Mortality among Rocky Shore Invertebrates across 100 km of Northeastern Pacific Coastline
Source: PLoS One. 2015 Jun 3;10(6):e0126280. doi: 10.1371/journal.pone.0126280 (PMC4454560; doi:10.1371/journal.pone.0126280)
Supplement: S3 Table — Table includes numbers of each species found in 2012 in large swath transects conducted at each site, with survey dates and area of coverage. (PDF) [file pone.0126280.s005.pdf]

S3 Table. Counts of *P. ochraceus* and *C. stelleri* in swath transects at 13 sites.

Site #	Site Name	Swath date	Area surveyed (m^2)	Inside kill zone[a]	Count *P. ochraceus*	Count *C. stelleri*
3	Moat Creek	09-Dec-12	1444	No	26	15
4	Iversen Point	16-Dec-12	1919	No	23	27
5	Serenisea	15-Dec-12	1857	Yes	66	25
8	Sea Ranch	17-Nov-12	1087	Yes	25	4
9	Fisk Mill Cove	16-Nov-12	831	Yes	28	0
10	Phillips Gulch	14-Dec-12	1788	Yes	71	28
14	Windermere Point	12-Nov-12	3736	Yes	12	3
16	Twin Coves	13-Dec-12	2080	Yes	36	19
17	Shell Beach	13-Nov-12	2799	Yes	0	0
19	Bodega Head	12-Dec-12	2357	Yes	39	2
22	Lifeboat House	10-Dec-12	2019	*	546	0
23	Palomarin	11-Dec-12	2317	No	143	0
24	Duxbury Reef	14-Nov-12	3134	No	30	0

Counts are of individuals found in 2012, in large swath transects with dates, area surveyed and location relative to zone of purple urchin loss.
[a] Kill zone defined by the loss of large urchin populations and *Leptasterias* sp.
*No pre-HAB urchin population
