# Supplementary material for: Patterns of Mass Mortality among Rocky Shore Invertebrates across 100 km of Northeastern Pacific Coastline
Source: PLoS One. 2015 Jun 3;10(6):e0126280. doi: 10.1371/journal.pone.0126280 (PMC4454560; doi:10.1371/journal.pone.0126280)
Supplement: S4 Table — Table includes numbers of Henricia sp. found in 0.25 m2 quadrats between 2012 and 2014, with locations, dates, and area surveyed. (PDF) [file pone.0126280.s006.pdf]

S4 Table. Counts of *Henricia* sp. in quadrats with locations, area and dates surveyed.

Site	Latitude (°N)	Longitude (°W)	Inside kill zone[a]	Date of first survey	Re-survey date	2012 count	2013-14 count	2012 total area (m^2)	2013-14 total area (m^2)
Van Damme SP	39.2803	123.8034	N	01-Jan-14	NA	NA	0	NA	37
Stornetta	38.9349	123.7262	N	31-Dec-13	NA	NA	15	NA	37
Arena Cove	38.9128	123.7108	N	12-Nov-12	02-Dec-13	2	44	5	21
Moat Creek	38.8801	123.6751	N	9-Dec-12	30-Dec-13	0	10	1	28
Iversen Point	38.8484	123.6467	N	16-Dec-12	02-Jan-14	0	7	20	37
Serenisea	38.7977	123.5728	Y	15-Dec-12	28-Jan-14	0	0	19	38
Del Mar Landing	38.7410	123.5082	Y	15-Nov-12	01-Dec-13	0	0	12	32
Sea Ranch	38.7001	123.4427	Y	17-Nov-12	29-Jan-14	1	0	14	34
Fisk Mill Cove	38.5969	123.3506	Y	16-Nov-12	03-Jan-14	0	0	11	35
Phillips Gulch	38.5865	123.3422	Y	14-Dec-12	30-Jan-14	0	0	16	32
Windermere Point	38.5250	123.2685	Y	12-Nov-12	05-Dec-13	0	0	14	32
Twin Coves	38.4586	123.1461	Y	13-Dec-12	31-Jan-14	0	0	20	40
Shell Beach	38.4178	123.1077	Y	13-Nov-12	06-Dec-13	0	0	12	40
Bodega Reserve	38.3197	123.0749	Y	16-Oct-12	04-Jan-14	0	0	1	26
Bodega Head	38.3034	123.0528	Y	12-Dec-12	04-Dec-13	0	0	17	37
McClures Beach	38.1823	122.9655	*	27-May-13	01-Feb-14	NA	0	20	28
Lifeboat House	37.9969	122.9796	*	10-Dec-12	15-May-14	0	0	15	38
Palomarin	37.9310	122.7495	N	11-Dec-12	29-Dec-13	1	1	18	38
Duxbury Reef	37.8931	122.7068	N	14-Nov-12	03-Dec-13	2	7	12	32
Pigeon Point	37.1827	122.3888	N	24-Jun-13	NA	NA	5	NA	18
Scott Creek	37.0442	122.2352	N	25-Jun-13	NA	NA	2	NA	19
Andrew Molera SP	36.2805	121.8634	N	23-Jun-13	NA	NA	2	NA	13

Animals found in 50 cm by 50 cm quadrats in surveys conducted between 2012 and 2014, with dates, area surveyed and site locations.
[a] Kill zone defined by the loss of large urchin populations and *Leptasterias* sp.
*No urchin population at this location prior to the mass mortality.
